# A novel on-line spatial-temporal k-anonymity method for location privacy protection from sequence rules-based inference attacks

**DOI:** 10.1371/journal.pone.0182232

**Published:** 2017-08-02

**Authors:** Haitao Zhang, Chenxue Wu, Zewei Chen, Zhao Liu, Yunhong Zhu

**Affiliations:** 1 School of Geographic and Biological Information, Nanjing University of Posts and Telecommunications, Nanjing, Jiangsu, China; 2 School of Telecommunications and Information Engineering, Nanjing University of Posts and Telecommunications, Nanjing, Jiangsu, China; University of Texas at San Antonio, UNITED STATES

## Abstract

Analyzing large-scale spatial-temporal k-anonymity datasets recorded in location-based service (LBS) application servers can benefit some LBS applications. However, such analyses can allow adversaries to make inference attacks that cannot be handled by spatial-temporal k-anonymity methods or other methods for protecting sensitive knowledge. In response to this challenge, first we defined a destination location prediction attack model based on privacy-sensitive sequence rules mined from large scale anonymity datasets. Then we proposed a novel on-line spatial-temporal k-anonymity method that can resist such inference attacks. Our anti-attack technique generates new anonymity datasets with awareness of privacy-sensitive sequence rules. The new datasets extend the original sequence database of anonymity datasets to hide the privacy-sensitive rules progressively. The process includes two phases: off-line analysis and on-line application. In the off-line phase, sequence rules are mined from an original sequence database of anonymity datasets, and privacy-sensitive sequence rules are developed by correlating privacy-sensitive spatial regions with spatial grid cells among the sequence rules. In the on-line phase, new anonymity datasets are generated upon LBS requests by adopting specific generalization and avoidance principles to hide the privacy-sensitive sequence rules progressively from the extended sequence anonymity datasets database. We conducted extensive experiments to test the performance of the proposed method, and to explore the influence of the parameter *K* value. The results demonstrated that our proposed approach is faster and more effective for hiding privacy-sensitive sequence rules in terms of hiding sensitive rules ratios to eliminate inference attacks. Our method also had fewer side effects in terms of generating new sensitive rules ratios than the traditional spatial-temporal k-anonymity method, and had basically the same side effects in terms of non-sensitive rules variation ratios with the traditional spatial-temporal k-anonymity method. Furthermore, we also found the performance variation tendency from the parameter *K* value, which can help achieve the goal of hiding the maximum number of original sensitive rules while generating a minimum of new sensitive rules and affecting a minimum number of non-sensitive rules.

## Introduction

The emergence of smartphones that are equipped with GPS receivers has made location-based services (LBS) increasingly popular. With the widespread development and adoption of LBS applications, location privacy has already been widely acknowledged as an important problem [1]. In recent years, location privacy of LBS applications has been the focus of many interdisciplinary research communities, and a number of technical methods have been proposed for protecting the privacy of LBS users [2]. Spatial-temporal k-anonymity has become a prominent method among privacy protection techniques because it is relatively simple and has a variety of applications. Most likely, LBS applications will continue to adopt spatial-temporal k-anonymity techniques in the future [3].

**Fig 1:**
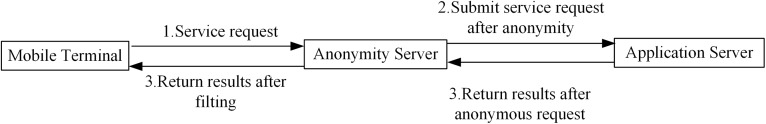
A distributed architecture implementation of spatial-temporal k-anonymity.

Generally, spatial-temporal k-anonymity is implemented in a distributed system architecture, as shown in Fig 1, where the application server for an LBS application can store and record large scale spatial-temporal k-anonymity datasets (hereinafter referred to as anonymity datasets). Then, the application server has an opportunity to apply sophisticated data mining techniques in order to discover knowledge from the anonymity datasets that can benefit many applications [4].

Technologies are intent-neutral, so the increased opportunity to discover knowledge also increases the risk of privacy violation. Privacy-sensitive knowledge discovered in this way can pose a significant threat to the privacy of individuals who are associated with the knowledge [5–7]. Unfortunately, because spatial-temporal k-anonymity and its series of optimization variants exist at the data-level, there are no countermeasures to prevent attacks based on privacy-sensitive knowledge mined from large-scale anonymity datasets.

Knowledge hiding (also known as knowledge cloaking) is one of the new emerging anti-attack approaches aimed at allowing the positive side of data mining to be utilized safely. However, while knowledge hiding can provide a solution for privacy protection problems in off-line publications, this approach cannot be applied directly to LBS applications. Knowledge hiding techniques cannot deal with the dynamic nature of LBS applications, which requires on-line privacy preservation techniques that differ from off-line techniques.

To overcome these challenges, first we defined a destination location prediction attack model based on privacy-sensitive sequence rules. We then proposed a novel on-line spatial-temporal k-anonymity method (*NOSTK*) designed to resist inference attacks based on privacy-sensitive knowledge mined from large-scale anonymity datasets generated by LBS continuous queries.

The remainder of this paper is organized as follows. Section *Related work* provides an overview of related work. In Section *Preliminaries*, we present necessary preliminary information and the basic concepts utilized in our research. In Section *Destination location prediction attack model*, we define privacy attacks formally. Then we introduce a specific class of privacy attacks known as the destination location prediction attack model, which is based on privacy-sensitive sequence rules. In Section *The proposed NOSTK method*, we present the proposed *NOSTK* method for defending against these attacks. In Section *Experiments and discussion*, we describe our comprehensive experiments and provide an analysis of the results. Section *Conclusion* concludes the paper.

## Related work

Location-based queries and trajectory publication are two main uses of LBS applications [8]. The former can be divided further into snapshot queries and continuous queries. Snapshot queries (e.g., “find the closest hotel”) require a user to report his/her current location to a service provider to find nearby points of interest. Continuous queries (e.g., “provide the locations of the closest gas stations while I am driving”) require the user to provide his/her location periodically. Various approaches to location privacy protection have been developed to address concerns about the security of location-based queries, including pseudonyms, anonymization, obfuscation, private information retrieval and differential privacy, among others. Spatial-temporal k-anonymity has become a prominent method for providing location privacy. In their work, [9–14] proposed a variety of diverse location privacy protection methods for snapshot queries. In [9], a spatial cloaking algorithm was proposed for mobile P2P environments, while [10] presented a spatial, identity, temporal, and activity conceptual model to achieve a location privacy protection method offering applicability and feasibility. The authors of [11] designed a density-based k-anonymization scheme that used a weighted adjacency graph to preserve a user’s privacy. In [12], the researchers developed a spatial-temporal location privacy preserving algorithm based on nearest neighbor search. In comparison, [13] designed a personalized spatial cloaking scheme, termed TTcloak, that could provide k-anonymity for a user's location privacy, 1-diversity for query privacy, and the desired size of the cloaking region for mobile users in an LBS, simultaneously. The authors of [14] observed that the existing methods required a large number of communication rounds between the user's device and the cloud server to answer a query, thereby creating high communication costs. In response, they proposed a k-anonymity algorithm, called Aman, to compute the cloaked area using a minimal number of communication rounds between the user and the cloud server [14].

Protecting user location privacy for continuous queries is more challenging than for snapshot queries because adversaries can use the spatial and temporal correlations in the user's sequence of location samples to infer the user's location information with a higher degree of certainty than for snapshot queries [15]. Various researchers, including but not limited to [16–20], proposed a series of location privacy protection methods for continuous queries. In their work, [16] presented a query perturbation-based scheme that protected query privacy for continuous queries in an LBS even when user identities were revealed. The research of [17] presented a query linking privacy preserving algorithm for continuous LBS queries by taking the user's velocity and acceleration similarity into consideration. In [18], the authors proposed a demand-aware location protection scheme for continuous LBS requests, allowing a user to customize not only location privacy but also Quality of Service requirements. The work of [19] offered a continuous query privacy-preserving framework in road networks, while [20] proposed a cloaking system model called anonymity of motion vectors that provided anonymity for spatial queries.

The location privacy protection that must be offered by trajectory publication applications needs to be stronger because they have larger granularity. Specifically, trajectory points taken from published trajectory datasets may be quasi-identifiers that can be linked to external information to identify a specified individual. Privacy-preserving trajectory publication techniques are divided into two main categories: methods of publishing independent location samples [21], and methods of publishing individual trajectories [22–27]. The authors of [21] derived a time-to-confusion criterion to characterize privacy in a locational data set, and proposed a disclosure control algorithm (called an uncertainty-aware path cloaking algorithm) that selectively revealed GPS samples to limit the maximum time-to-confusion for all vehicles. In [22], a data suppression technique was devised that prevented linking attacks based on quasi-identifiers, while keeping the posted data as accurate as possible. The research of [23] explored a concept of k-anonymity based on co-localization that exploited the inherent uncertainty of the moving object's whereabouts. The authors of [24] demonstrated a generalization-based approach to address privacy issues regarding the identification of individuals in static trajectory datasets, while [25] proposed a greedy clustering-based approach for anonymizing trajectory data in which the privacy requirements of moving objects were not necessarily the same. In [26], the researchers examined a distributed and efficient strategy that adopted the k-anonymity privacy model and used the scalable MapReduce paradigm, which allowed their method to find quasi-identifiers in larger amounts of data. In addition, [27] proposed a segment clustering-based privacy preserving algorithm that could prevent re-clustering attacks against the characteristics of large-scale trajectory databases.

It is worth mentioning that differential privacy, as a new robust privacy protection model, has been recognized as a milestone in fields of privacy data protection and extensively studied by researchers in recent years. Some researchers have also proposed differential privacy methods for Location-based queries[28–31] and trajectory publication [32–34]. In their work, [28] developed a real-time framework that guaranteed differential privacy for individual users and releases accurate data for research purposes. In [29], the authors proposed a generalized version of differential privacy for location-based systems which exploited geographical in distinguish ability to protect user privacy within a certain radius. The research of [30] presented a predictive differentially-private mechanism for mobility traces by exploiting correlations in traces to provide a prediction function that tries to guess the new location based on the previously reported locations. The work of [31] offered a ϵ-differential privacy method which can be applied to an infinite stream of “events”. The authors of [32] designed by using hierarchical reference system a strong privacy protection method with the form of e-differential privacy for trajectory publication, while [33] presented an efficient data-dependent yet differentially private transit data sanitization approach based on a hybrid-granularity prefix tree structure. In addition, [34] proposed a (e, d)-differentially private interesting geographic location pattern mining approach motivated by the sample-aggregate framework.

However, all the location privacy protection methods mentioned above, whether it is a weak privacy mode (e.g., spatial-temporal k-anonymity) or more robust privacy model (e.g., differential privacy), there is a common problem: they function at the data level, so they cannot deal with inference attacks based on privacy-sensitive knowledge mined from large scale anonymity datasets (Although [34] takes into account geographic location patterns, it only ensures the availability of interesting patterns and does not consider how to deal with attacks based on sensitive patterns). For example, if an adversary secures sequential patterns that reflect the movement of collective LBS users, and the sequential patterns are involved in privacy-sensitive regions, then for any individual who satisfies the conditions of the patterns–i.e., for any individual whose current or historical location intersects with the locations of the privacy-sensitive sequential patterns–the future or historical location of that individual can be inferred.

The existing approaches to privacy-sensitive knowledge sanitization that hide sensitive patterns and association rules were designed mainly for privacy protection data publishing applications. These techniques adopted the strategies of distortion and blocking, which could prohibit the leakage of sensitive knowledge in the published dataset while avoiding any downgrades to the effectiveness of the dataset. The approaches involved three main types of knowledge: association rules, classification models, and sequence patterns [7]. Approaches involving association rules have been the most abundant [35–37]. The work developed in [35] summarized available algorithms and schemes for hiding association rules, and described an advanced decrease support of right hand side items of rule cluster algorithm. In [36], the researchers proposed a model for hiding sensitive association rules that was implemented with a Fast Hiding Sensitive Association Rule algorithm using the Java Eclipse framework. In addition, [37] proposed a new distortion-based method that could hide sensitive rules by removing some items in a database to reduce the support or confidence of sensitive rules below specified thresholds. However, the existing knowledge hiding methods (e.g., association rules, classification models, and sequence patterns [7]) did not perform well for LBS applications, mainly because LBS applications have an inherently dynamic nature. Specifically, the hiding privacy-sensitive knowledge can be rediscovered from anonymity datasets recorded by an application server (a potential attacker), as the anonymity datasets will be dynamically updated with the LBS system running continuously. In this paper, we proposed the *NOSTK* method to solve the problem.

## Preliminaries

In this section, we introduce the basic concept of spatial-temporal k-anonymity, along with the sequential rules mined from large scale anonymity datasets that are processed by spatial-temporal k-anonymity.

### Spatial-temporal k-anonymity

Spatial-temporal k-anonymity is a branch of the k-anonymity method, which is an obfuscation technique [38]. The basic principle of the technique involves cloaking a requestor’s identification as well as any precise information about the requestor’s position and the time of the request. Spatial-temporal k-anonymity and its optimized versions are applied widely in LBS snapshot queries and continuous queries. To better understand the follow-up analysis of anonymity datasets, we present examples of anonymity datasets for an LBS snapshot query and an LBS continuous query.

An anonymity dataset for an LBS snapshot query is defined as follows:

SnAS=⟨UP,CR,TC⟩, (1)

where *UP* = ⟨*U*_1_,*U*_2_,…,*U*_*k*_⟩ represents a set of *k* user pseudonyms, *CR* = ⟨*Cell*_1_,*Cell*_2_,…,*Cell*_*m*_⟩ represents a cloaking region that includes *m* grid cells enclosing the locations of the k users, and *TC* = ⟨*TI*_1_,*TI*_2_,…,*TI*_*n*_⟩ represents temporal cloaking with n time intervals of equal duration. Here, the time intervals ⟨*TI*_1_,*TI*_2_,…,*TI*_*n*_⟩ provide very little temporal information, i.e., *SnAS* is a temporally-ordered sequence without a specified time. An example of *SnAS* is *SnAS* = ⟨⟨*U*_1_,*U*_2_,*U*_3_,*U*_4_,*U*_5_,*U*_6_,*U*_7_,*U*_8_,*U*_9_,*U*_10_,*U*_11_⟩,⟨*Cell*_22_,*Cell*_23_,*Cell*_33_⟩,⟨1⟩⟩.

Based on the definition of an anonymity dataset for a snapshot query, we define an anonymity dataset for an LBS continuous query as shown:

$$CoAS=\left\langle SnAS_{1},SnAS_{2},\ldots ,SnAS_{s}\right\rangle ,$$ (2)

where *SnAS*_*i*_(1 ≤ *i* ≤ *s*) represents an anonymity dataset for a snapshot query. Some examples include: *CoAS* = ⟨*SnAS*_1_,*SnAS*_2_,*SnAS*_3_,*SnAS*_4_⟩,

$$\begin{array}{l} SnAS_{1}=\left\langle \begin{array}{l} \left\langle U_{11},U_{12},U_{13},U_{14},U_{15},U_{16},U_{17}\right\rangle ,\\ \left\langle Cell_{22},Cell_{23},Cell_{33}\right\rangle ,\left\langle 1\right\rangle \end{array}\right\rangle ,SnAS_{2}=\left\langle \begin{array}{l} \left\langle U_{3},U_{4},U_{5},U_{6},U_{8},U_{14},U_{15},U_{16}\right\rangle ,\\ \left\langle Cell_{15},Cell_{16},Cell_{26}\right\rangle ,\left\langle 2\right\rangle \end{array}\right\rangle ,\\ SnAS_{3}=\left\langle \begin{array}{l} \left\langle U_{11},U_{12},U_{13},U_{14},U_{22},U_{23},U_{24}\right\rangle ,\\ \left\langle Cell_{27},Cell_{37},Cell_{38}\right\rangle ,\left\langle 3\right\rangle \end{array}\right\rangle ,SnAS_{4}=\left\langle \begin{array}{l} \left\langle U_{1},U_{2},U_{7},U_{8},U_{9},U_{10},U_{14}\right\rangle ,\\ \left\langle Cell_{112},Cell_{211},Cell_{212}\right\rangle ,\left\langle 4\right\rangle \end{array}\right\rangle . \end{array}$$

Since only the spatial and temporal properties of anonymity datasets are the focus of this paper, an anonymity dataset for an LBS continuous query simply can be equivalent to a sequence of cloaking regions, denoted as follows:

SCR=⟨⟨CR1,TC1⟩,⟨CR2,TC2⟩,…,⟨CRs,TCs⟩⟩. (3)

In the case of the anonymity dataset *CoAS* = ⟨*SnAS*_1_,*SnAS*_2_,*SnAS*_3_,*SnAS*_4_⟩, the corresponding sequence of the cloaking regions is as follows:

SCR=⟨⟨CR1,⟨1⟩⟩,⟨CR2,⟨2⟩⟩,⟨CR3,⟨3⟩⟩,⟨CR4,⟨4⟩⟩⟩,

where $\begin{array}{l} CR1=\left\langle Cell_{22},Cell_{23},Cell_{33}\right\rangle ,CR2=\left\langle Cell_{15},Cell_{16},Cell_{26}\right\rangle ,\\ CR3=\left\langle Cell_{27},Cell_{37},Cell_{38}\right\rangle ,CR4=\left\langle Cell_{112},Cell_{211},Cell_{212}\right\rangle \end{array}$

Furthermore, we defined ⟨*Cell*_22_,*Cell*_15_,*Cell*_38_,*Cell*_112_⟩ as a sequence of query grid cells (*SQGC*) corresponding to the sequence of cloaking region, where *Cell*_22_,*Cell*_15_,*Cell*_38_,*Cell*_112_ continuously contain the requestor’s locations.

### Sequence rules mined from sequences of the LBS cloaking regions

We define a sequence rule mined from sequences of the cloaking regions [39,40]as follows:

$$SeR=\left\{\left\langle gc_{1}\Rightarrow gc_{2}\to gc_{3}\to ,\ldots ,\to gc_{n}\right\rangle ,\left\langle Supp,Conf\right\rangle \right\},$$ (4)

where *gc*_*i*_ (1 ≤ *i* ≤ *n*) represents a grid cell in which an LBS user issues a request, and *gc*_*i*_ occurs before *gc*_*i*+1_. *gc*_1_ and *gc*_2_ → *gc*_3_ →,⋯,→ *gc*_*n*_ are defined as the antecedent and descendant of *SeR*, respectively; *Supp*,*Conf* are defined as the support and confidence of *SeR*, respectively; and *n* is the length of *SeR*.

A sequence rule (*SeR*) indicates that, if an LBS user issues an anonymous request in grid cell *gc*_1_, then with the confidence (*Conf*), he/she will present successively n-1 anonymous requests in grid cells *gc*_2_,*gc*_3_,…,*gc*_*n*_, respectively. In addition, if the last element *gc*_*n*_ of the descendant *gc*_2_ → *gc*_3_ →,⋯,→ *gc*_*n*_ intersects with a privacy-sensitive region (*PSR*) (for example, a military restricted area), the sequence rule is called a privacy-sensitive sequence rule (*PSSR*). Otherwise, the rule is called a non-privacy-sensitive sequence rule (*nPSSR*).If *n* = 2, we call the rule a *SingleRule*; otherwise, if *n* > 2, it is a *MultiRule*. In this paper, we focused on *SingleRules*. In fact, a *MultiRule* is a combination of multiple *SingleRules*. For example, for a *MultiRule A* = {⟨*gc*_1_ ⇒ *gc*_2_ → *gc*_*3*_ →,⋯,→*gc*_*n*_⟩,⟨*Supp*,*Conf*⟩}, it can be obtained by combining multiple *SingleRules*: *B*_1_ = {⟨*gc*_1_ ⇒ *gc*_2_⟩,⟨*Supp*,*Conf*⟩}, *B*_2_ = {⟨*gc*_1_ ∧ *gc*_2_ ⇒ *gc*_3_⟩,⟨*Supp*,*Conf*⟩},…, *B*_*n*−1_ = {⟨*gc*_1_ ∧ *gc*_2_ ∧⋯∧ *gc*_*n*−1_ ⇒ *gc*_*n*_⟩,⟨*Supp*,*Conf*⟩}.

Then, the *NOSTK* method proposed in this paper can also be extended to *MultiRules*. Table 1 presents a sample of *SingleRules* mined from anonymity datasets generated by LBS continuous queries.

**Table 1:** A sample of *SingleRules*.

No.	*SeR*	*Conf*	*Supp*
1	A = >B	0.2	0.8
2	A = >C	0.5	0.75
3	A = >D	0.2	0.85
4	B = >C	0.7	0.95
5	B = >E	0.3	0.95
6	C = >D	0.1	0.9
7	C = >F	0.6	0.8
8	D = >F	0.9	0.87
9	E = >F	0.8	0.82

## Destination location prediction attack model

Providing a suitable definition of an attack model for privacy violation is vital in order to design a corresponding countermeasure. An attack model generally consists of four components [41]: (1) Target private information. (2) Messages exchanged during provision of service. (3) Background knowledge. (4) Inference abilities available to an adversary. In the next sections, we will give the specific implementation of these four components in the context of LBS.

### Target private information

The target private information that an attacker expects to obtain is the probability that an LBS user, moving from his/her current position (e.g., a grid cell) will arrive at a privacy-sensitive region. Based on that probability, an attacker may infer more privacy-sensitive information, such as a user’s religion, political orientation, or sexual orientation.

### Messages exchanged during provision of service

As shown in Fig 1, the basic workflow of message exchanges between components of an LBS system can be described as follows:

1. A mobile terminal sends a request to an anonymity server.
2. The anonymity server generates an anonymity dataset and sends it to an application server.
3. The application server performs a spatial-temporal computation based on the anonymity dataset, and the application server returns a rough result to the anonymity server.
4. The anonymity server filters the rough result to get an actual result, which it returns to the mobile terminal.

We can see from this process that messages exchanged between an anonymity server and a potential attacker, i.e., an application server, consist mainly of anonymity datasets and corresponding anonymous query results.

### Background knowledge

The skills of an adversary may vary strongly depending on the context. Potential attackers have various levels of ability to obtain different types of domain-specific background knowledge, which they can use to threaten individual privacy. Consequently, different assumptions about the background knowledge sought by an attacker entail distinct defense strategies [42]. The counter measure(s) to be adopted must change significantly once the assumptions about the background knowledge change [6].

In this paper, in order to ensure the robustness of our presented attack model and its associated countermeasure, we focus on one specific type of background knowledge: privacy-sensitive sequence rules mined from large-scale historical anonymity datasets generated by LBS continuous queries.

### Inference abilities available to an adversary

The inference ability available to an adversary is the capability of the adversary to get the target private information by combining messages exchanged with background knowledge. Specifically, the potential attacker attempts to get a probability value for an LBS user moving from his/her current position (e.g., a grid cell) and arriving at a privacy-sensitive region. This information is gained by combining a current anonymity dataset generated for the user and the privacy-sensitive sequence rules mined from large-scale historical anonymity datasets generated for a large number of users.

The simulated attack scenario works as follows: (1) The attacker mines sequential rules from large-scale sequences of cloaking regions, and matches the rules with privacy-sensitive regions specified by the attacker in order to get all of the privacy-sensitive sequence rules. (2) The attacker obtains a current anonymity dataset transmitted by the anonymity server, and learns through some illegal means (e.g., by eavesdropping on the communication channels or by using a collusion attack) that a specific user is most likely included in the current anonymity dataset. (3) The attacker acquires a probability value that the user will move from his/her current position and arrive at a privacy-sensitive region, by matching the grid cell corresponding to the user’s current position with the antecedent grid cell of the privacy-sensitive sequence rules.

Considering the sequential rules in Table 1, we assume a region intersecting with the grid cell *F* as a privacy-sensitive region. Then, sequence rules (*C* ⇒ *F*,0.9), (*D* ⇒ *F*,0.8), (*E* ⇒ *F*,0.6) are all privacy-sensitive sequence rules. Next, we assume that the potential attacker obtains an anonymity dataset from an anonymity server *SnAS* = ⟨⟨*U*_1_,*U*_2_,*U*_3_,*U*_4_,*U*_5_,*U*_6_,*U*_7_,*U*_8_,*U*_9_,*U*_10_,*U*_11_⟩,⟨*C*,*G*,*H*⟩,⟨1⟩⟩, and learns, for example, that a user named Bob is included in the anonymity datasets. Finally, the potential attacker can use this information about Bob, along with the privacy-sensitive sequence rule (*C* ⇒ *F*,0.9), to make the inference that there is 0.3 (= $\frac{1}{3}\times 0.9$) probability as the cloaking region contains three grid cells *C*, *G*, *H*, which means that Bob will move from the grid cell and arrive at the grid cell *F* with 0.3 probability.

## The proposed NOSTK method

In this section, we provide details about the proposed NOSTK defense method and its development.

### System architecture

In this research, we developed a novel on-line spatial-temporal k-anonymity (*NOSTK*) method to deal with the destination location prediction attack described above, taking into account the on-line characteristics of LBS applications. The system architecture of this anti-attack technique includes two stages: off-line analysis and on-line application.

In the off-line analysis stage, a cloaking server, in a manner similar to an application server, stores anonymity datasets generated by LBS continuous queries. The cloaking server also mines sequence rules from large-scale anonymity datasets, and gathers privacy-sensitive sequence rules by joining the mined spatial sequence rules with the privacy-sensitive regions specified.

In the on-line application stage, with dynamic awareness of privacy-sensitive sequence rules, the anonymity server continuously generates anonymity datasets based on the demand for anonymous services by LBS users until all privacy-sensitive sequence rules are hidden. In other words, the privacy-sensitive sequence rules cannot be mined from the final sequence database of anonymity datasets under the same or higher parameter settings (i.e., thresholds of confidence and support). The final sequence database consists of the original sequence database and the new anonymity datasets generated by the *NOSTK* method. Using this approach, the destination location prediction attack based on privacy-sensitive sequence rules can be eliminated.

### Avoidance and generalization

The support value and confidence value are two significance measures of a sequence rule. A simple and effective way to hide privacy-sensitive sequence rules is to decrease these two measures. Because sequence rules are constructed using statistical significance, hiding privacy-sensitive sequence rules may result in modification of a portion of the non-privacy-sensitive sequence rules [43]. The ideal solution is to limit disclosure of privacy-sensitive sequence rules completely by reducing their significance, leaving the significance of non-privacy-sensitive sequence rules unaltered or minimally affected. However, it has been proven that finding an exact optimal solution is *NP-hard* [44, 45]. Alternatively, a heuristic-based approximation approach provides an efficient solution that is able to hide privacy-sensitive sequence rules as well as balance utilization of non-privacy-sensitive sequence rules.

We adopted a heuristic-based strategy to generate new on-line anonymity datasets with awareness of privacy-sensitive sequence rules. Here, two types of principles must be followed: avoidance and generalization. The avoidance principle is always used in the process of generating anonymity datasets on-line. According to this principle, when generating a cloaking region of anonymity datasets, it is essential to avoid creating a region consisting of nearby *PSSR* grid cells that are included in privacy-sensitive sequence rules.

The generalization principle can be divided into three sub-types according to characteristics of the current grid cell that contains the requestor's location. (1) If the current grid cell is not a *PSSR* grid cell, i.e., it is an *nPSSR* grid cell, the minimum generalization is used. In other words, the generated cloaking region should contain as few nearby *nPSSR* grid cells as possible. (2) If the current grid cell involves privacy-sensitive regions, the grid cell is referred to as a *PSR* grid cell, and the maximum generalization is used. Since a privacy-sensitive region is the most important factor for generating privacy-sensitive sequence rules, a generated cloaking region should contain as many nearby *nPSSR* grid cells as possible. (3) If the current grid cell is a *PSSR* grid cell, but not a *PSR* grid cell, normal generalization is used, i.e., the cloaking region can be generated by just avoiding inclusion of *PSSR* grid cells located near the current grid cell.

The impact of these principles on privacy-sensitive sequence rules is clear. On the one hand, privacy-sensitive sequence rules can be hidden rapidly because the avoidance principle and the maximum generalization principle can help to minimize support for the rules. On the other hand, the utilization of non-privacy-sensitive sequence rules can be maintained also, as the normal generalization principle and the minimum generalization principle are adopted. Specifically, the normal generalization principle ensures minimal effect on non-privacy-sensitive sequence rules, while the minimum generalization principle mainly offsets the effect of the maximum generalization principle on non-privacy-sensitive sequence rules.

### Implementation of the NOSTK method

**Fig 2:**
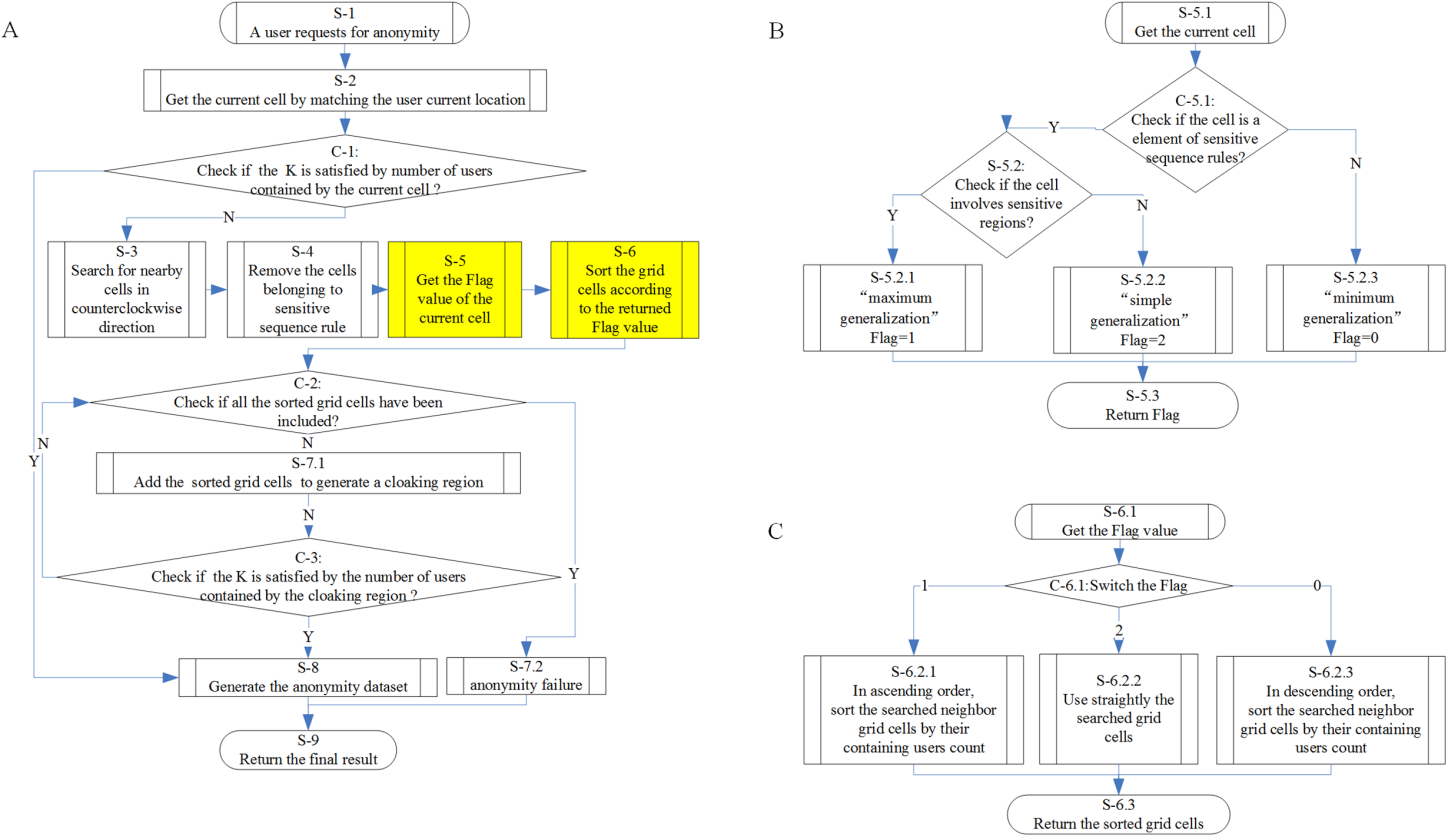
Workflow for generating anonymity datasets based on the NOSTK method. (A)The main flow chart. (B)The sub-flow chart for checking the current grid cell and selecting a generalization principle. (C)The sub-flow chart for sorting searched neighbor grid cells based on selected generalization principles.

The implementation process for the *NOSTK* method is shown in Fig 2. For clarity, Fig 2A is the main process flow chart, and Fig 2B and 2C are two sub-flow charts. In Fig 2A:

**Step S-1** responds to an LBS user’s request for anonymity.

**Step S-2** searches the LBS user’s current location, and gets the current grid cell containing the location through the use of spatial matching.

**Step C-1** checks whether *K* is satisfied by counting other users contained by the current cell. If the value of *K* is satisfied (true), then the process moves to Step P-8, at which point an anonymity dataset is generated consisting of the current cell and its contained users, or else the process continues to Step S-3.

**Step S-3** searches grid cells near the current grid cell in a counterclockwise direction.

**Step S-4** filters out any *PSSR* grid cells from the result. This step is the implementation of the avoidance principle.

**Step S-5** checks whether the current grid cell belongs to *PSSR* grid cells and/or *PSR* grid cells, and selects a specific generalization principle. Details of this part of the process are shown in Fig 2B.

**Step S-6** sorts searched neighbor grid cells based on selected generalization principles. Specifically, an ascending order, a descending order, and a normal order correspond to the maximum generalization principle, the minimum generalization principle, and the normal generalization principle, respectively. Details of this part of the process are shown in Fig 2C.

**Step C-2** checks whether all sorted grid cells have been used. If the result is false (i.e., all sorted grid cells have not been used), the process moves to **Step S-7.1** and **Step C-3** to add sorted grid cells one by one to generate a cloaking region and simultaneously to check whether *K* is satisfied by the number of users contained by the cloaking region. In contrast, if the result for Step C-2 is true (i.e., all sorted grid cells have been used), then the process goes to **Step S-8** to generate an anonymity dataset consisting of searched cells and their contained users. If all sorted grid cells have been used and the anonymity dataset has not been generated, then the process moves to **Step S-7.2** to generate a failure result.

As can be seen from these steps, the generalization principle is achieved by generating cloaking regions using sorted grid cells. Specifically, once setting the same *K* value threshold, by using the ascending grid cells, the descending grid cells and the grid cells that are not sorted, the cloaking region generated contains the largest number of grid cells (i.e., the cloaking region is maximized), the least number of grid cells (i.e., the cloaking region is minimized) and the middle number of grid cells (i.e., the cloaking region is normalized), respectively. This process will be confirmed in the following *Running Example* section.

**Step S-9** returns a final result.

The pseudo-codes for the algorithm of Fig 2A are shown as Algorithm 1 below, which is the main algorithm. The pseudo-codes for Step S-4 and for Step S-5 are shown as the subroutines labeled Algorithm 2 and Algorithm 3 respectively. The pseudo-codes for Step S-6 through Step S-8 are shown as the subroutine labeled Algorithm 4.

**Algorithm 1:** *AS*: *NOSTK* (*Cell*_*cur*_,*Cells*_*PSR*_,*Cells*_*PSSR*_,*K*)

      **Input:** *Cell*_*cur*_ represents a current grid cell that contains the location of an LBS user. *Cells*_*PSR*_ represents *PSR* grid cells; *Cells*_*PSSR*_ represents *PSSR* grid cells; *K* represents the least number of users among the generated anonymity dataset.

      **Output:** *AS* represents the generated anonymity dataset.

1.    if (*Cell*_*cur*_∙*GetUsersCount*()≥*K*)

2.    *AS*⋅*add*(*Cell*_*cur*_⋅*GetUsers*,*Cell*_*cur*_);

3.    else {

4.    *Cells*_*Avoid*_ = *RemoveSensi*(*Cell*_*cur*_⋅*Near*,*Cells*_*PSSR*_);

5.    *Flag*_*generalization*_ = *GetGenerFlag*(*Cell*_*cur*_,*Cells*_*PSR*_,*Cells*_*PSSR*_);

6.    *AS* = *GenerateAS*(*Cell*_*cur*_,*Cells*_*Avoid*_,*Flag*_*generalization*_);}

7.    return *AS*;

**Algorithm 2:** *Cells*_*Avoid*_: *RemoveSensi*(*Cell*_*cur*_⋅*Near*,*Cells*_*PSSR*_)

      **Input:** *Cell*_*cur*_⋅*Near* represents all grid cells adjacent to *Cell*_*cur*_. *Cells*_*PSSR*_ is the same parameter as in Algorithm 1.

      **Output:** *Cells*_*Avoid*_ represents the grid cells that are filtered *PSSR* grid cells from *Cell*_*cur*_⋅*Near*.

1.    *Cells*_*Near*_ = *Cell*_*cur*_⋅*Near*;

2.    for each *Cell*∈*Cells*_*Near*_

3.        if (*Cell*∈*Cells*_*PSSR*_) *continue*;

4.        else *Cells*_*Avoid*_⋅*add*(*Cell*);

5.    return *Cells*_*Avoid*_;

**Algorithm 3:** *Flag*_*generalization*_: *GetGenerFlag*(*Cell*_*cur*_,*Cells*_*PSR*_,*Cells*_*PSSR*_)

    **Input:** *Cell*_*cur*_, *Cells*_*PSR*_, *Cells*_*PSSR*_ are the same parameters as in Algorithm 1.

    **Output:** *Flag*_*generalization*_ represents a flag indicating the adoption of a generalization principle.

1.  if (*Cell*_*cur*_∈*Cells*_*PSSR*_)

2.  if (*Cell*_*cur*_∈*Cells*_*PSR*_)

3.  *Flag*_*generalization*_ = 1;

4.  else *Flag*_*generalization*_ = 2;

5.  else *Flag*_*generalization*_ = 0;

**Algorithm 4:** *AS*: *GenerateAS* (*Cell*_*cur*_,*Cells*_*Avoid*_,*Flag*_*generalization*_,*K*)

    **Input:** *Cell*_*cur*_ and *K* are the same parameters as in Algorithm 1. *Cells*_*Avoid*_ is the same parameter as in Algorithm 2, and *Flag*_*generalization*_ is the same parameter as in Algorithm 3.

    **Output:** *AS* is the same parameter as in Algorithm 1.

1.  if (*Flag*_*generalization*_ = 0) *Cells*_*sort*_ = *DescSort*(*Cells*_*Avoid*_);

1.  else if (*Flag*_*generalization*_ = 1) *Cells*_*sort*_ = *AsceSort*(*Cells*_*Avoid*_);

2.  else if (*Flag*_*generalization*_ = 2) *Cells*_*sort*_ = *Cells*_*Avoid*_;

3.  *UsersCount* = *Cell*_*cur*_⋅*GetUsersCount*();

4.  *UP*⋅*add*(*Cell*_*cur*_⋅*GetUsers*); *CR*⋅*add*(*Cell*_*cur*_);

5.  for each *Cell*∈*Cells*_*sort*_{

6.  *UP*⋅*add*(*Cell*⋅*GetUsers*); *CR*⋅*add*(*Cell*);

7.  *UsersCount* + = *Cell*⋅*GetUsersCount*();

8.  if (*UsersCount* ≥ *K*){

9.  *AS*⋅*add*(*UP*,*CR*); return *AS*;}

10.  return *Null*;

### Running example

**Fig 3:**
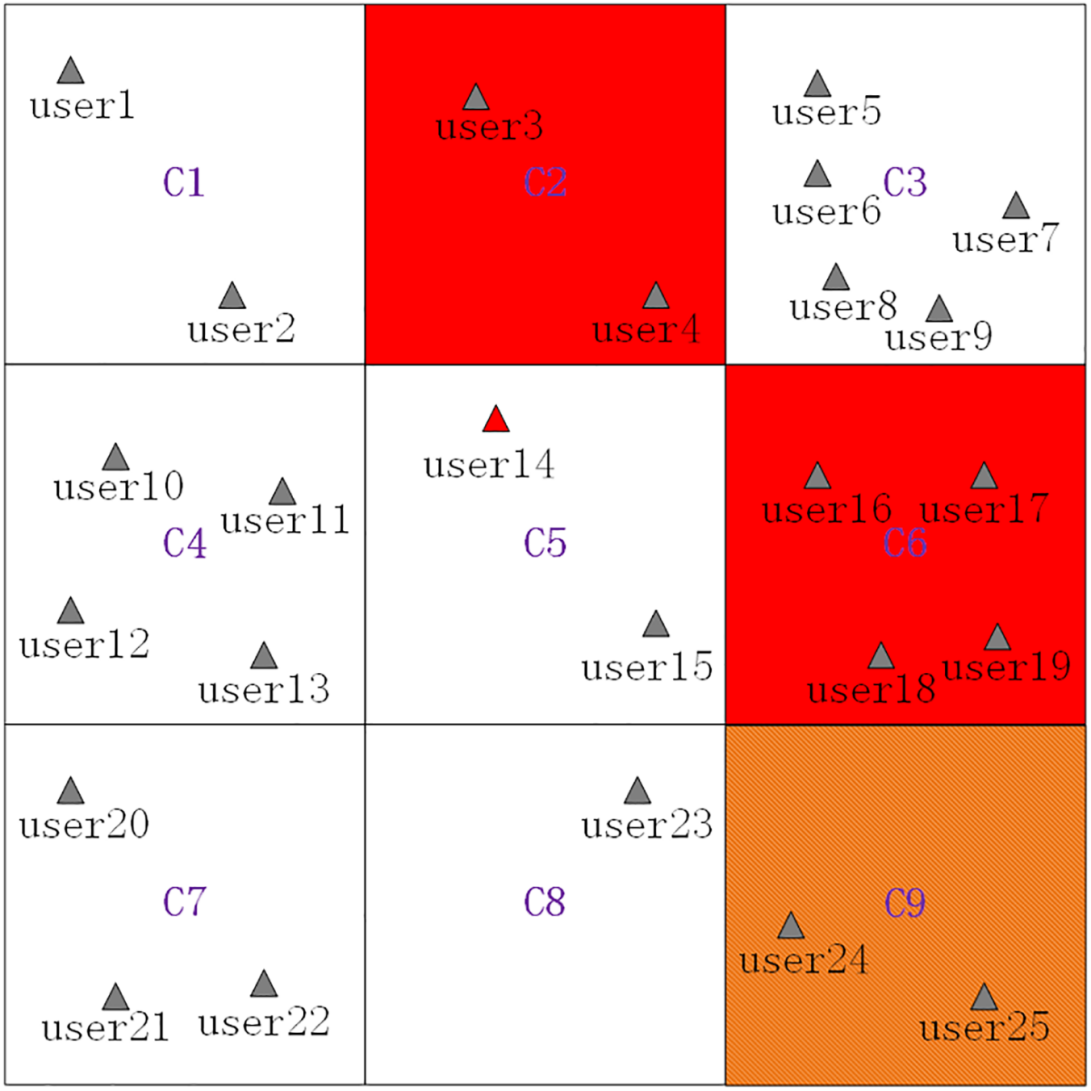
A scenario that the current grid cell is an *nPSSR* grid cell.

We use an example as follows to better explain how these algorithms are executed. Fig 3 presents the spatial distribution of 25 LBS users in a certain period, where *user*_14_ contained at grid cell *C*_5_ is the requester of an anonymity service, that is, *C*_5_ is the current grid cell. *C*_2_ and *C*_6_ are privacy-sensitive region grid cells (*PSR* grid cells, which are filled with red color), and *C*_6_ ⇒ *C*_9_ is a privacy-sensitive sequence rule achieved by a cloaking server. Then, *C*_9_ is a privacy-sensitive sequence rule grid cell (*PSSR* grid cell, which is filled with brownish yellow color) but not a *PSR* grid cell, and *C*_5_ is an *nPSSR* grid cell. Furthermore, the minimum generalization principle will be used in the process of generating a cloaking region, that is, the generated cloaking region will contain as few *nPSSR* grid cells as possible.

**Fig 4:**
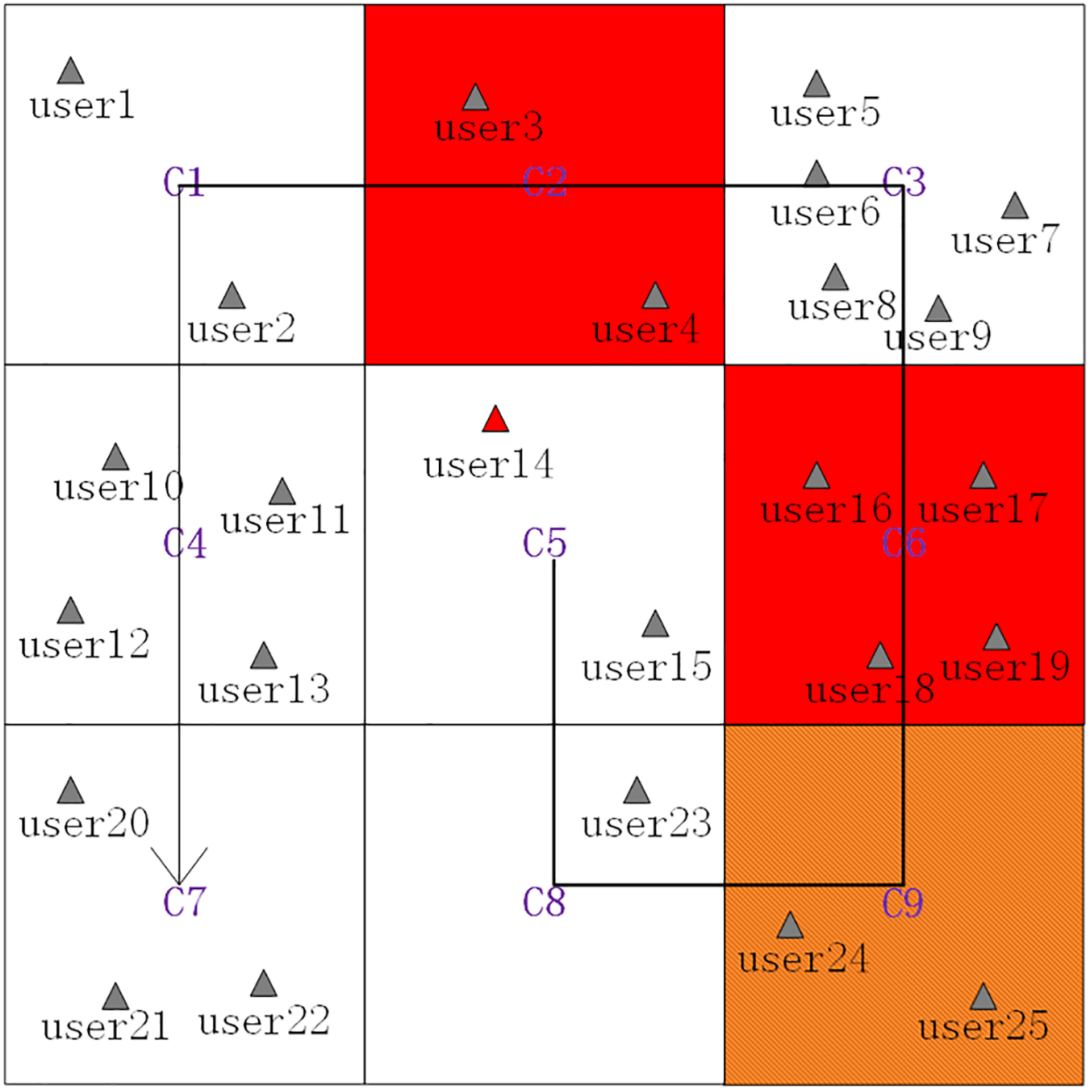
Searching grid cells near *C*_5_ in a counterclockwise direction.

**Fig 5:**
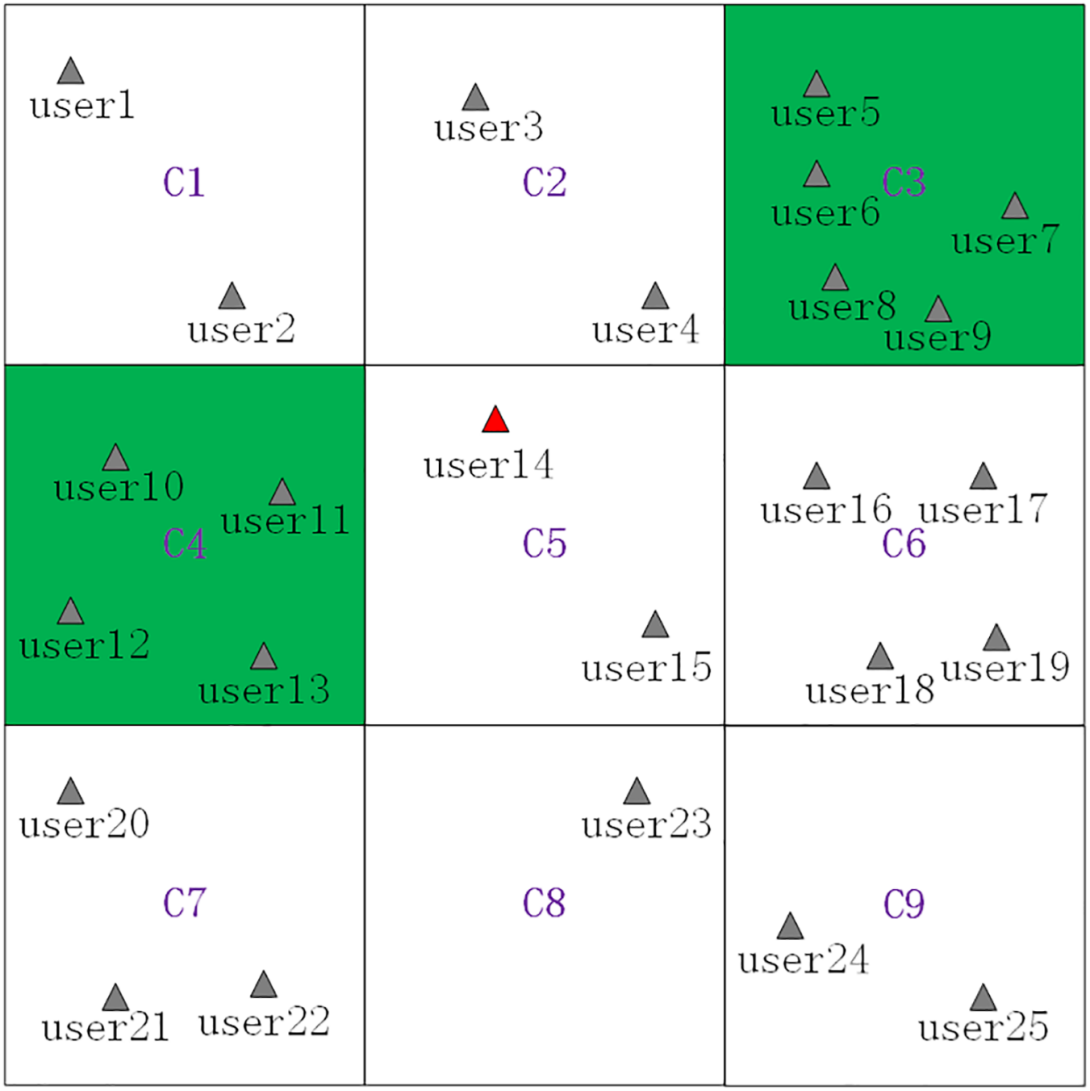
The generated cloaking region based on the avoidance principle and the minimum generalization principle.

The basic process of generating a cloaking region for *user*_14_ is as follows, where the *K* value is set to 8:

1. Searching grid cells grids near *C*_5_ in a counterclockwise direction (shown as Fig 4) to get searched grid cells ⟨*C*_1_,*C*_2_,⋯,*C*_9_⟩.
2. Removing the *PSSR* grid cell(*C*_9_) and the *PSR* grid cell(*C*_2_ and *C*_6_) from the searched grid cells ⟨*C*_1_,*C*_2_,⋯,*C*_9_⟩ to get filtered grid cells ⟨*C*_1_,*C*_3_,*C*_4_,*C*_5_,*C*_7_,*C*_8_⟩.
3. Sorting the filtered grid cells in a descending order by their contained users counts to get sorted grid cells ⟨*C*_3_,*C*_4_,*C*_7_,*C*_1_,*C*_5_,*C*_8_⟩, which are shown as Table 2.
4. Adding the sorted grid cells ⟨*C*_3_,*C*_4_,*C*_7_,*C*_1_,*C*_5_,*C*_8_⟩ one by one to generate a cloaking region and simultaneously checking whether *K = 8* is satisfied by the number of users contained by the cloaking region. Specifically, when *C*_3_ and *C*_4_ are added, the contained users counts is 9 which satisfies the set *K* value, and the finally generated cloaking region is ⟨*C*_3_,*C*_4_⟩, which consists of grid cells filled with green color, and shown as Fig 5.

**Table 2:** The grid cells sorted in a descending order.

Grid cell	User count
C3	5
C4	4
C7	3
C1	2
C5	2
C8	1

**Fig 6:**
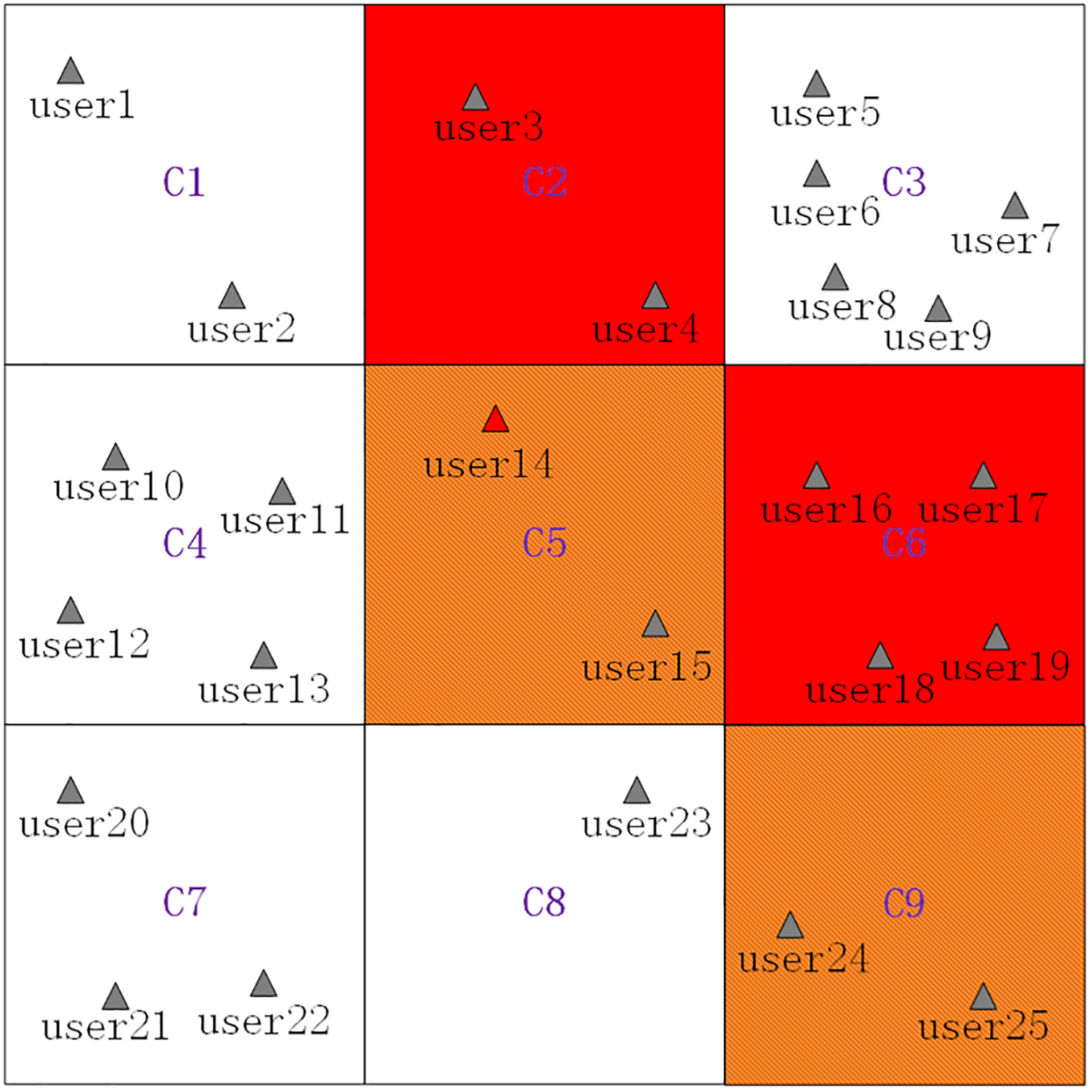
A scenario that the current grid cell is a *PSSR* grid cells but not a *PSR* grid cell.

**Fig 7:**
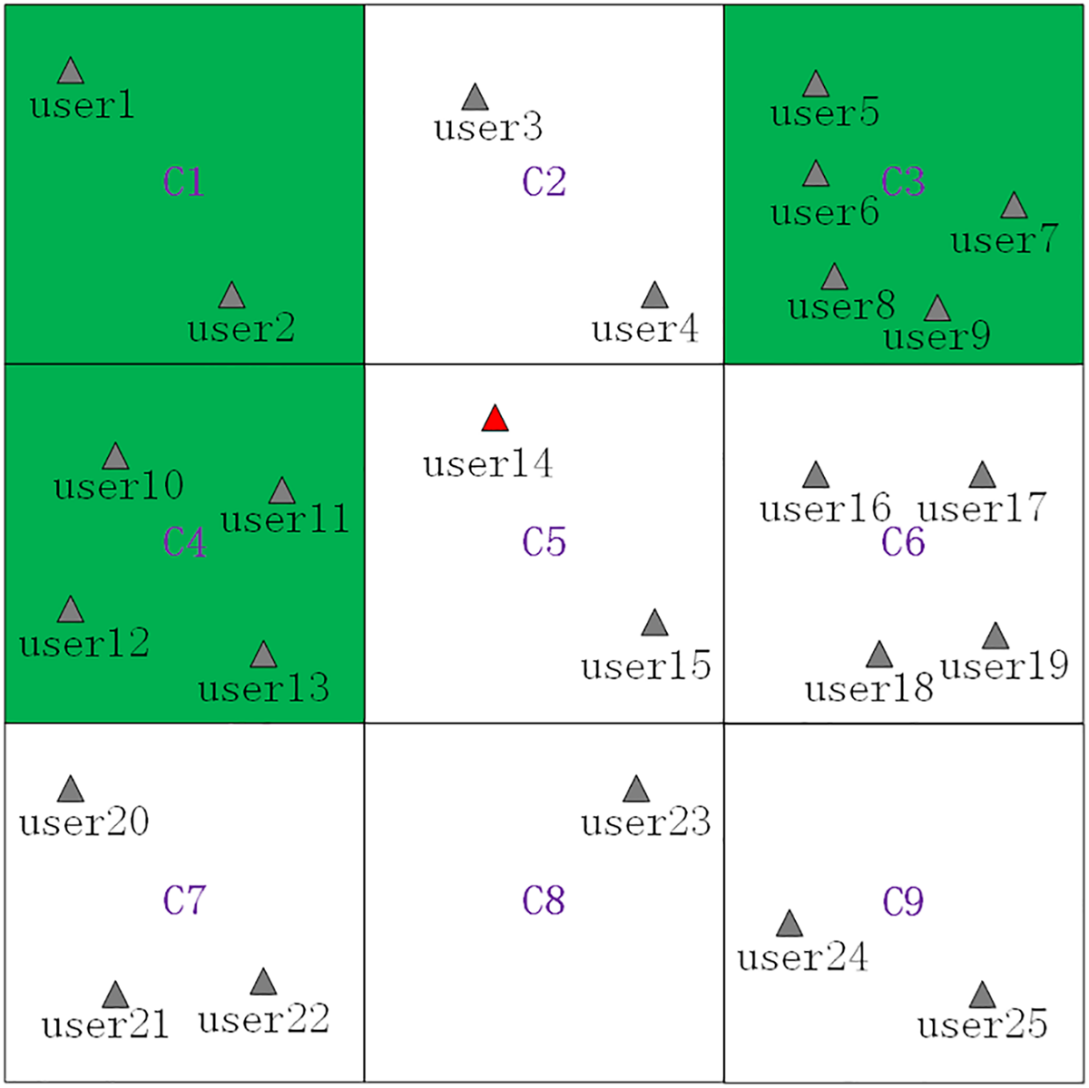
The generated cloaking region based on the avoidance principle and the normal generalization principle.

Next, we give a **scenario** where the current grid *C*_5_ is a *PSSR* grid cells but not a *PSR* grid cell. This **scenario** is shown as Fig 6, which is similar to Fig 3 except that there is another privacy-sensitive sequence rule *C*_6_ ⇒ *C*_5_. Then, the normal generalization principle will be used in the process of generating a cloaking region, and the basic process of generating a cloaking region for *user*_14_ is as follows, where the *K* value is also set to 8:

1. Removing the *PSSR* grid cells (*C*_9_ and *C*_5_) and the *PSR* grid cells (*C*_2_ and *C*_6_) from the searched grid cells ⟨*C*_1_,*C*_2_,⋯,*C*_9_⟩ to get the filtered grid cells ⟨*C*_1_,*C*_3_,*C*_4_,*C*_7_,*C*_8_⟩, which are shown as Table 3.
2. Adding filtered grid cells ⟨*C*_1_,*C*_3_,*C*_4_,*C*_7_,*C*_8_⟩ one by one to generate a cloaking region and simultaneously checking whether *K = 8* is satisfied by the number of users contained by the cloaking region. Specifically, when *C*_1_, *C*_3_ and *C*_4_ are added, the contained users counts is 11 and satisfies the set *K* value, and the finally generated cloaking region is ⟨*C*_1_,*C*_3_,*C*_4_⟩, which is shown as Fig 7.

**Table 3:** The grid cells filtered out *PSSR* grid cells and *PSR* grid cells.

Grid cell	User count
C1	2
C3	5
C4	4
C7	3
C8	1

**Fig 8:**
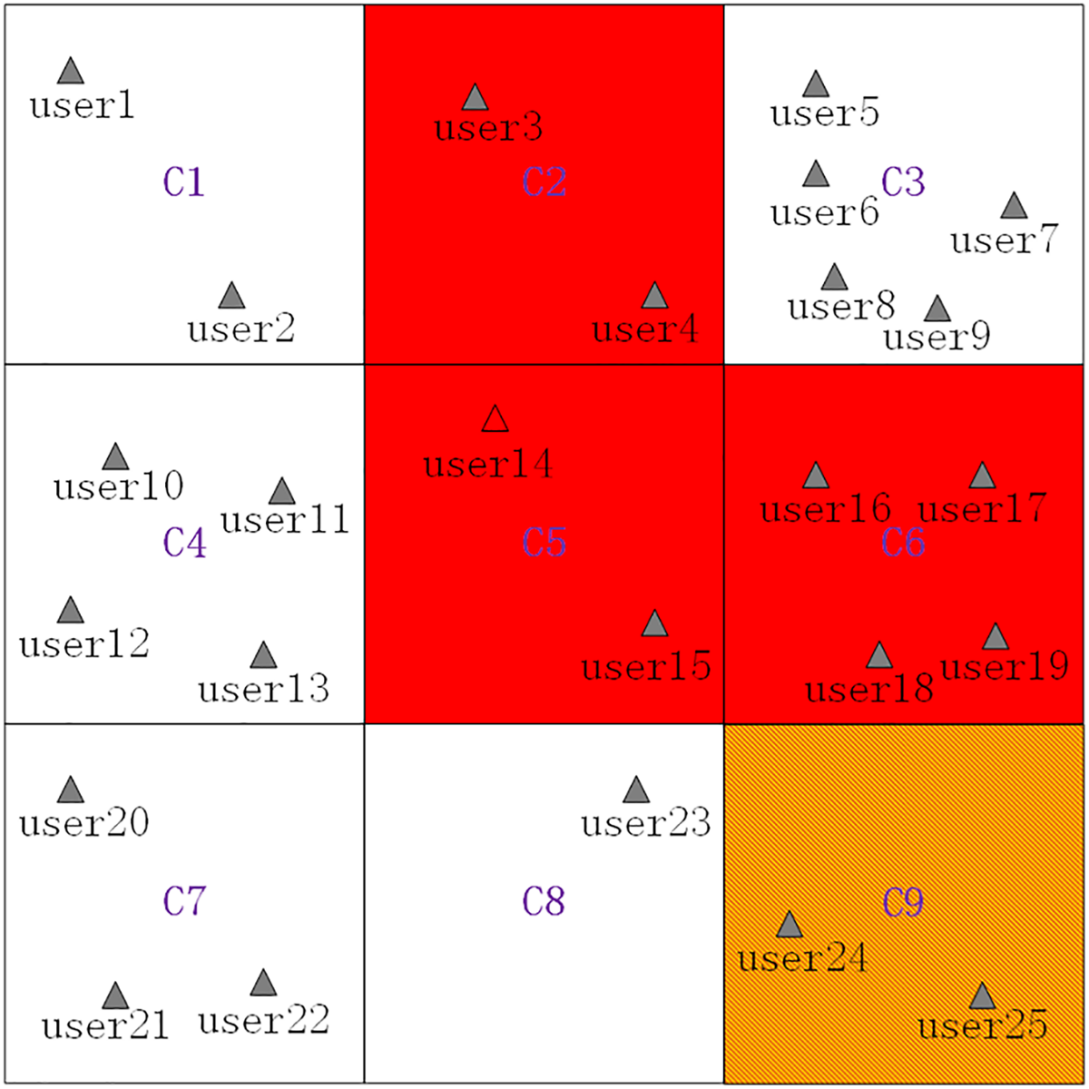
A scenario that the current grid cell is a *PSR* grid cell.

**Fig 9:**
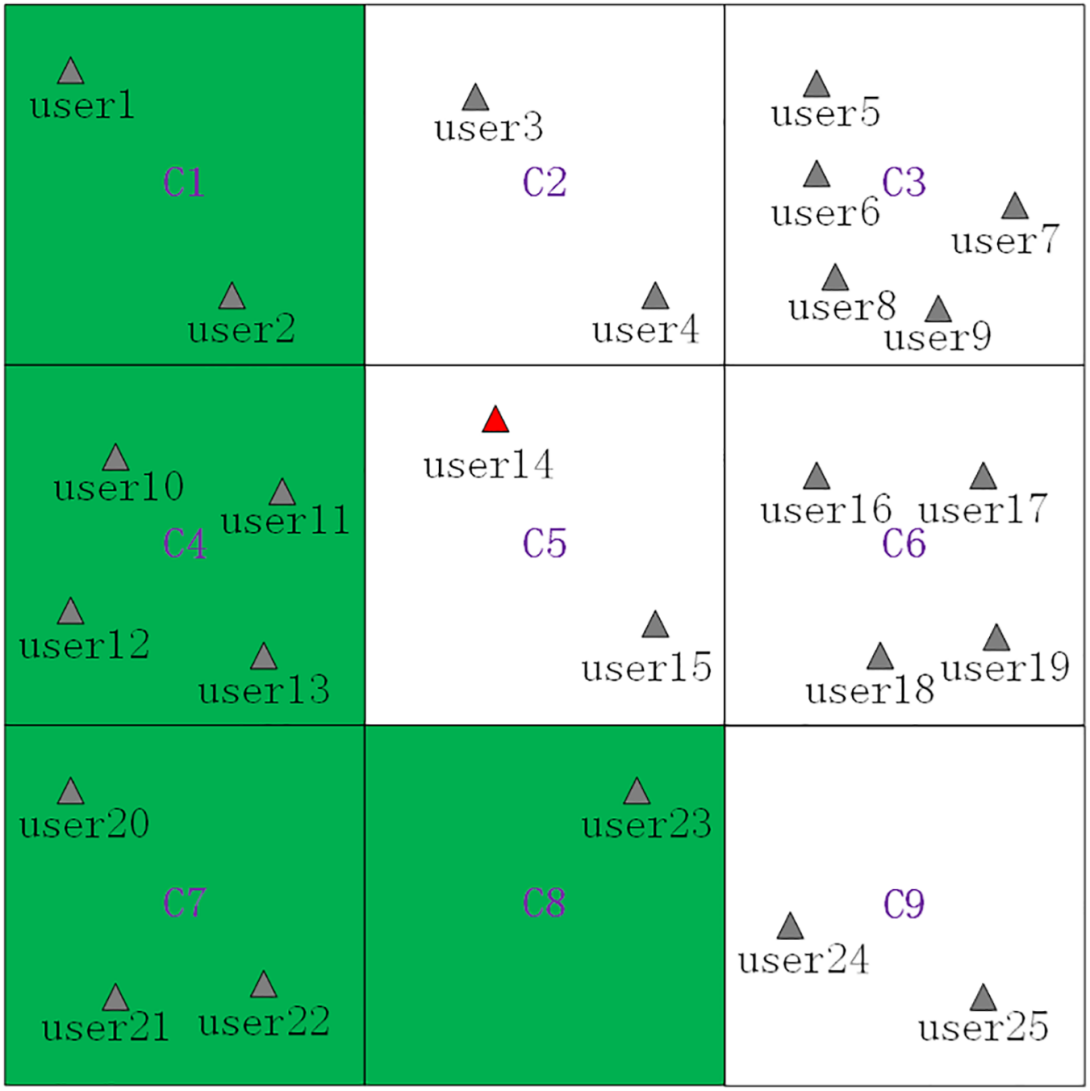
The generated cloaking region based on the avoidance principle and the maximum generalization principle.

Finally, we give a **scenario** where the current grid *C*_5_ is a *PSR* grid cell, which is shown as Fig 8, where *C*_2_, *C*_5_ and *C*_6_ are *PSR* grid cells, and *C*_9_ is a privacy-sensitive sequence rule grid cell as the privacy-sensitive sequence rule *C*_6_ ⇒ *C*_9_. Then, the maximum generalization principle will be used in the process of generating a cloaking region, and the basic process of generating a cloaking region for *user*_14_ is as follows, where the *K* value is continually set to 8:

1. Removing the *PSSR* grid cell (*C*_9_) and the *PSR* grid cells(*C*_2_, *C*_5_ and *C*_6_) from the searched grid cells ⟨*C*_1_,*C*_2_,⋯,*C*_9_⟩ to get the filtered grid cells ⟨*C*_1_,*C*_3_,*C*_4_,*C*_7_,*C*_8_⟩.
2. Sorting the filtered grid cells in an ascending order by their contained users counts to get the sorted grid cells ⟨*C*_8_,*C*_1_,*C*_7_,*C*_4_,*C*_3_⟩, which are shown as Table 4.
3. Adding the sorted grid cells ⟨*C*_8_,*C*_1_,*C*_7_,*C*_4_,*C*_3_⟩ one by one to generate a cloaking region and simultaneously checking whether *K = 8* is satisfied by the number of users contained by the cloaking region. Specifically, when *C*_8_, *C*_1_, *C*_7_ and *C*_4_ are added, the contained users counts is 11 which satisfies the set *K* value, and the finally generated cloaking region is ⟨*C*_8_,*C*_1_,*C*_7_,*C*_4_⟩, which is shown as Fig 9.

**Table 4:** The grid cells sorted in an ascending order.

Grid Cell	User count
C8	1
C1	2
C7	3
C4	4
C3	5

## Experiments and discussion

In this section, we describe the experiments we used to test the performance stability of our proposed method and to find the performance variation tendency from the parameter *K* value. We also provide a discussion of the results.

### Data preparation

The following sections describe the steps taken to prepare the data.

#### Simulated sequences of cloaking regions for LBS continuous queries

In order to test the performance stability of our proposed method and explore the influence from the parameter *K* value, we used a traditional spatial-temporal k-anonymity method (*tSTK*) [46] to simulate 81 batches of sequences of cloaking regions with 9 different *K* values (10~18). Specifically, for each *K* value, we simulated 9 batches of sequences of cloaking regions, and for each batch, we sampled 500 users’ trajectories to simulate LBS continuous queries and generate approximately 500 sequences of cloaking regions. Finally, we obtained approximately 40,500 sequences of cloaking regions. Detailed information for the simulated datasets is shown in Table 5.

**Table 5:** Batches of sequences of cloaking regions with K = 10~18.

K	No	Num of *SCR*	K	No	Num of *SCR*	K	No	Num of *SCR*	K	No	Num of *SCR*	K	No	Num of *SCR*
10	1	479	11	1	479	12	1	479	13	1	479	14	1	479
	2	488		2	488		2	488		2	488		2	488
	3	489		3	489		3	489		3	489		3	489
	4	488		4	488		4	488		4	488		4	488
	5	485		5	485		5	485		5	485		5	485
	6	484		6	484		6	484		6	484		6	484
	7	492		7	492		7	492		7	492		7	492
	8	489		8	489		8	489		8	489		8	489
	9	488		9	488		9	488		9	488		9	488
15	1	479	16	1	478	17	1	478	18	1	478			
	2	488		2	488		2	488		2	488			
	3	489		3	489		3	489		3	489			
	4	488		4	487		4	487		4	487			
	5	485		5	485		5	485		5	485			
	6	484		6	484		6	484		6	484			
	7	492		7	492		7	492		7	492			
	8	489		8	488		8	488		8	488			
	9	488		9	488		9	488		9	488			

#### Sequence rules mined from simulated datasets and specified privacy-sensitive rules

Using an approach developed in the previous literature [40–47],we obtained 81 batches of sequence rules by mining from 81 batches of sequences of cloaking regions in Table 5, and setting *MinSup* = 2% and *MinConf* = 10% (The number of sequence rules will vary greatly with the set support threshold and the confidence threshold. If there are too few or too many sequence rules, it is difficult to test the performance of the algorithm. Based on the number and distribution of simulated sequences of cloaking regions, we set MinSup as 2% and MinConf as 10%). Furthermore, we specified privacy-sensitive sequence rules that involve the most frequent grid cells for partial batches of sequence rules. Detailed information for the simulated datasets is shown in Table 6.

**Table 6:** Sequence rules mined from Table 5 and specified privacy-sensitive rules.

K	No	Num of *SeR*	Num of *PSSR*	K	No	Num of *SeR*	Num of *PSSR*	K	No	Num of *SeR*	Num of *PSSR*
10	1	39	28	11	1	37	25	12	1	36	24
	2	30	21		2	25			2	24	
	3	36	29		3	37			3	32	
	4	28	23		4	30			4	29	
	5	24	27		5	24			5	26	
	6	37	33		6	36			6	39	
	7	25	20		7	28			7	25	
	8	39	29		8	38			8	40	
	9	27	24		9	26			9	28	
13	1	34	24	14	1	36	25	15	1	34	25
	2	26			2	22			2	21	
	3	33			3	32			3	32	
	4	29			4	30			4	26	
	5	27			5	23			5	20	
	6	36			6	39			6	35	
	7	26			7	26			7	25	
	8	43			8	39			8	37	
	9	30			9	27			9	27	
16	1	34	25	17	1	34	25	18	1	31	23
	2	20			2	21			2	19	
	3	33			3	34			3	29	
	4	27			4	26			4	23	
	5	22			5	24			5	24	
	6	36			6	38			6	32	
	7	26			7	30			7	28	
	8	36			8	36			8	32	
	9	26			9	26			9	21	

#### Data expansion using the *tSTK* method and the *NOSTK* method

As described in *System Architecture* section, the cloaking server off-line exploits large-scale anonymity datasets to get privacy-sensitive sequence rules, and then on-line uses the *NOSTK* method to generate anonymity datasets based on the demand for anonymous services by LBS users. In order to highlight the performance of the *NOSTK* method, we also assumed that the cloaking server uses the *tSTK* method to generate anonymity datasets, and simulated the generating two processes by two different data expansion strategies.

The data expansion strategy for using the *tSTK* method for data expansion combines incrementally different batches of sequences of cloaking regions with the same *K* value and with different *K* values. For each batch of privacy-sensitive sequence rules with *K* = 10 in Table 6, we incrementally combined its corresponding batch of sequences of cloaking regions in Table 5 with other batches of sequences of cloaking regions in Table 5 to get several incremental combinations.

For example, for batch No. 5 of privacy-sensitive sequence rules, we combined incrementally batches No. 5 sequences of cloaking regions in Table 5, with batches Nos. 1~4, and batch Nos. 6~9 of sequences of cloaking regions in Table 5, and obtained 8 batches of incremental combinations. Likewise, for batches Nos. 2~10 of privacy-sensitive sequence rules with *K* = 10 in Table 6, we also were able to get 8 batches of incremental combinations. In total, we gathered 72 batches of incremental combinations.

In contrast, the data expansion strategy for using the *NOSTK* method regenerates sequences of cloaking regions for all sequences of query grid cells that correspond to batches of sequences of cloaking regions.

For example, for batch No. 1 consisting of privacy-sensitive sequence rules with *K* = 15 in Table 6, first we collected sequences of query grid cells corresponding to 8 other batches of sequences of cloaking regions with *K* = 15 in Table 5 (specifically, batches Nos. 2~9). Then for each grid cell among the sequences of query grid cells, based on the *NOSTK* method, we regenerated 8 batches of sequences of cloaking regions and got 8 batches of incremental combinations. In total, we also gathered 72 batches of incremental combinations. Similar to the *tSTK* method, for 9 batches consisting of No. 1 of privacy-sensitive sequence rules with different *K* values in Table 6 (*K* = 10~18), we were able to get 72 batches of incremental combinations as well. It is worth noting that Batch No.1 privacy-sensitive sequence rules with K = 10 was also included.

For the three batches of 72 incremental combinations, from which we mined sequence rules by using the approach developed in the previous literature [40–47] as mentioned above, and setting the same parameters (i.e., *MinSup* = 2% and *MinConf* = 10%), we compared the mined rules with their corresponding original privacy-sensitive sequence rules.

To highlight the performance advantages of the *NOSTK* method, we compared it with the *tSTK* method on three performance evaluation metrics: the ratio of hiding sensitive rules (i.e., the percentage of privacy-sensitive sequence rules that were not discovered from a final sequence database), the ratio of newly generated sensitive rules (i.e., the percentage of privacy-sensitive sequence rules that were newly discovered from a final sequence database), and the ratio of non-sensitive rules variation (i.e., the percentage of non-privacy-sensitive sequence rules that were not discovered and newly discovered from a final sequence database). Hiding sensitive rules would be expected, while generating new sensitive rules and non-sensitive rules variation would be viewed as two side effects of the process.

### Experiment 1: Performance comparison between the *NOSTK* and the *tSTK* methods on datasets with the same parameter *K* value

**Fig 10:**
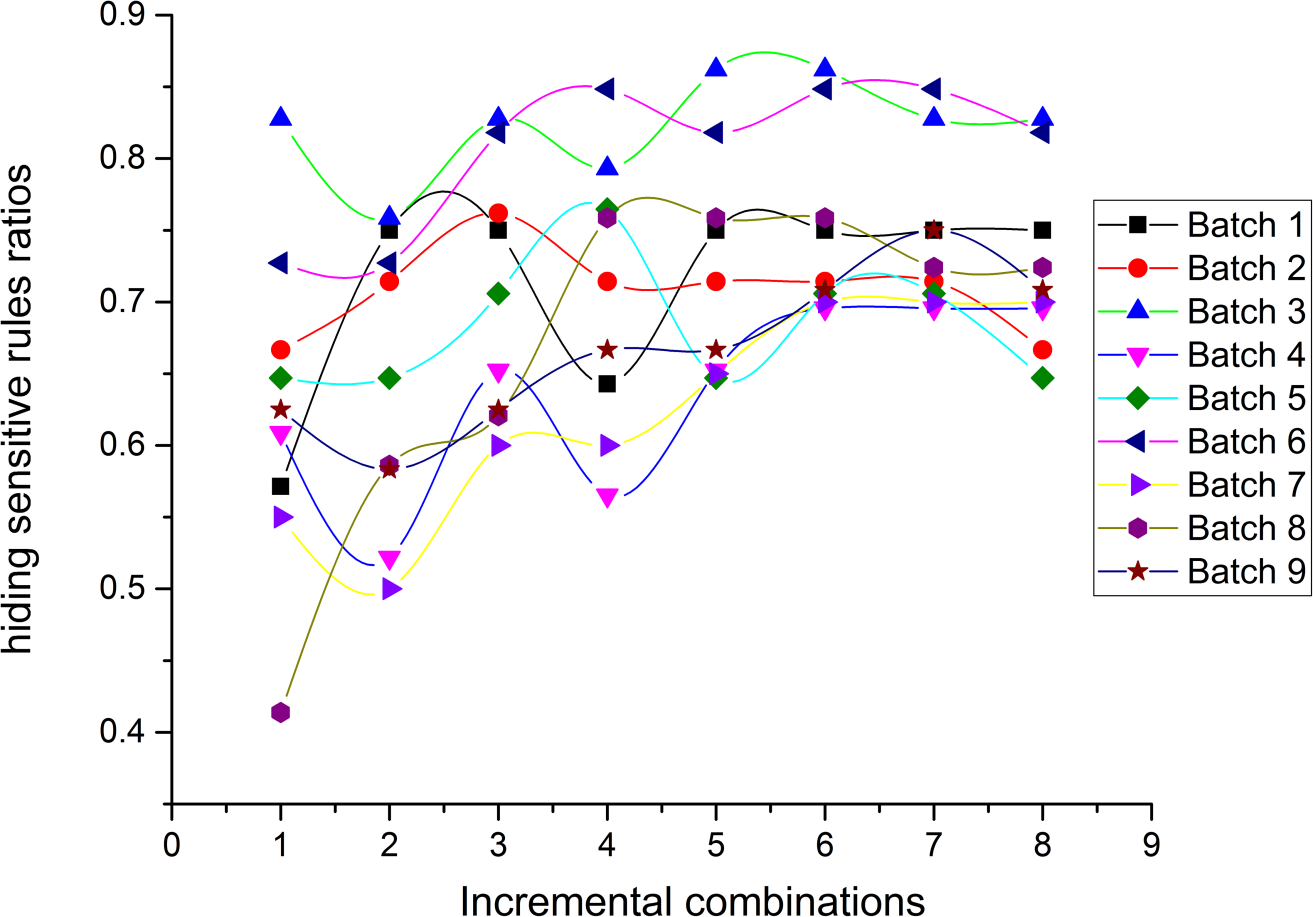
Hiding sensitive rules ratios change with data expansion using the *NOSTK* method.

**Fig 11:**
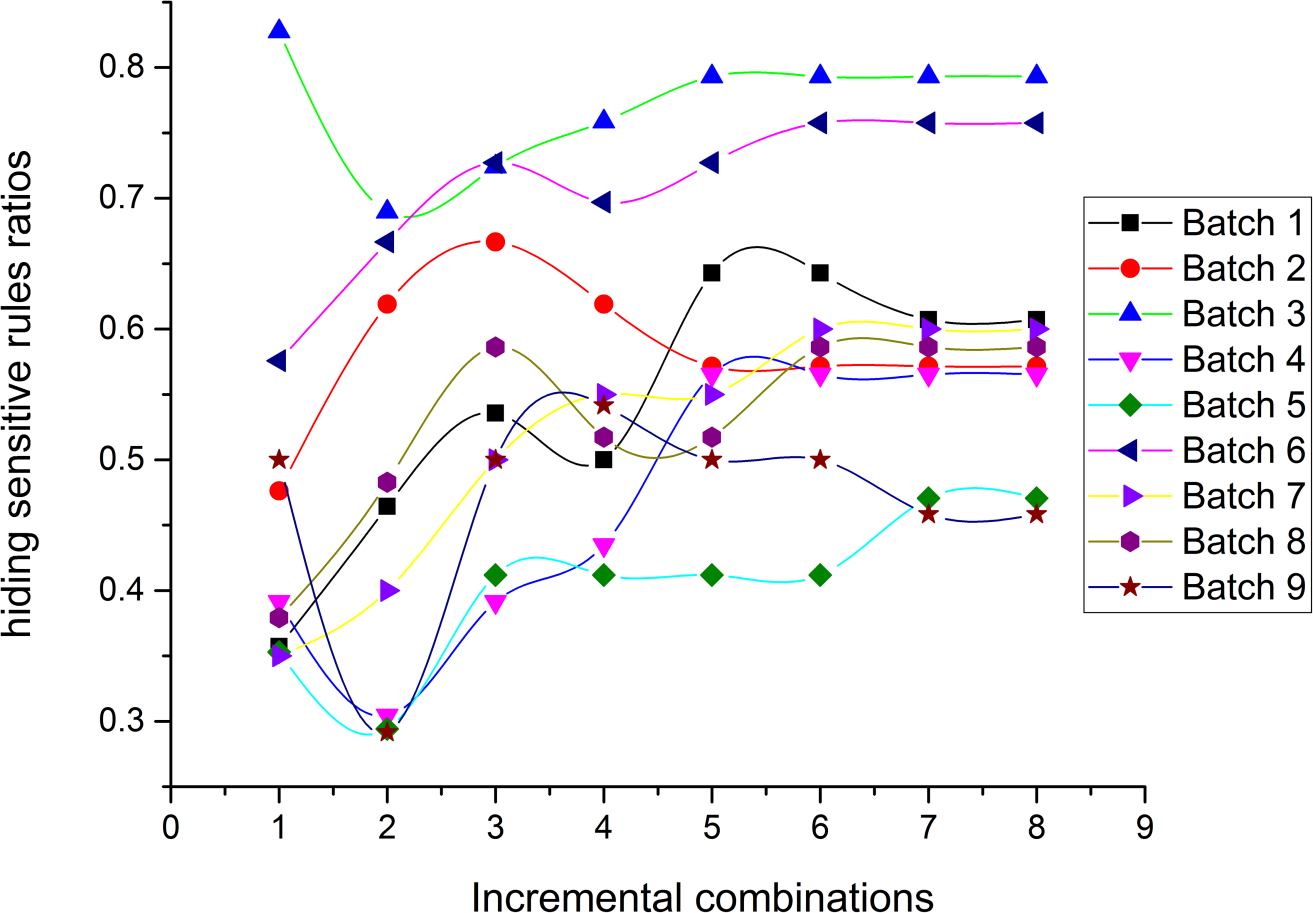
Hiding sensitive rules ratios change with data expansion using the *tSTK* method.

This experiment compared the performance of the *NOSTK* method with the *tSTK* method using the two performance evaluation metrics mentioned previously. In this experiment, we tested the sequence rules mined from two batches of 72 incremental combinations with the same parameter value (*K* = 10). Figs 10 and 11 present the results in terms of hiding sensitive rules ratios, for the *NOSTK* method and the *tSTK* method, respectively.

The following can be seen from the comparison: (1) Overall, for different batches of privacy-sensitive sequence rules with the same *K* value, the ratios for hiding sensitive rules based on the *NOSTK* method varied less and maintained a higher range of average values than the ratios based on the *tSTK* method. In particular, for the same batch of data, and the same incremental combination, none of the ratios for hiding sensitive rules based on the *NOSTK* method were less than the values based on the *tSTK* method.(2) As combinations increased, ratios for hiding sensitive rules based on the *NOSTK* method quickly tended toward a higher range of values. Specifically, after an incremental combination was up to 5, for all batches of privacy-sensitive sequence rules the range using the *NOSTK* method was [0.647058824–0.862068966], while the range for batches using the *tSTK* method was [0.411764706–0.793103448]. These results demonstrated that the *NOSTK* method was more stable in terms of hiding sensitive rules ratios, and could hide almost all sensitive rules more quickly than the *tSTK* method.

**Fig 12:**
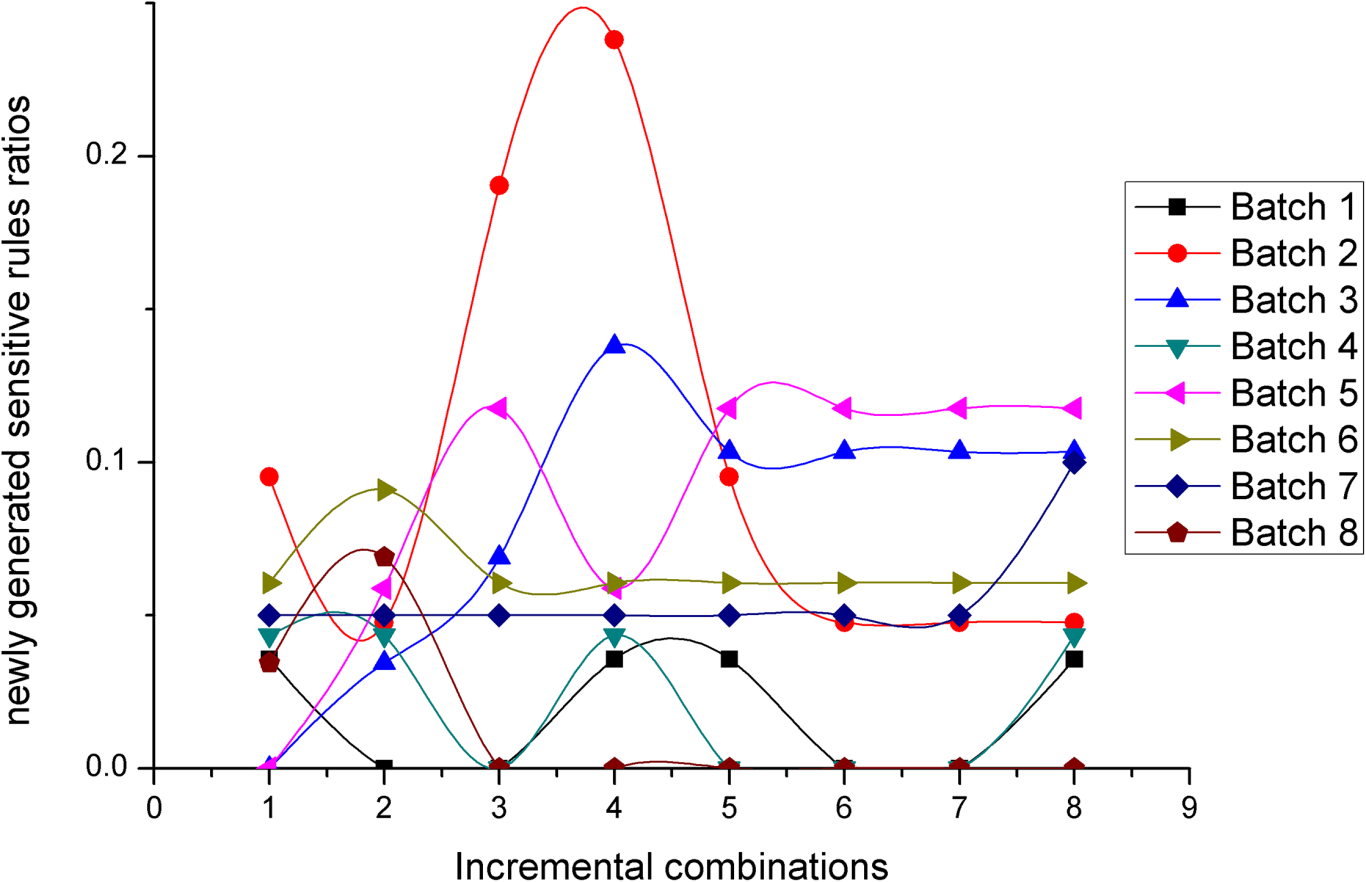
Newly generated sensitive rules ratios change with data expansion using the *NOSTK* method.

**Fig 13:**
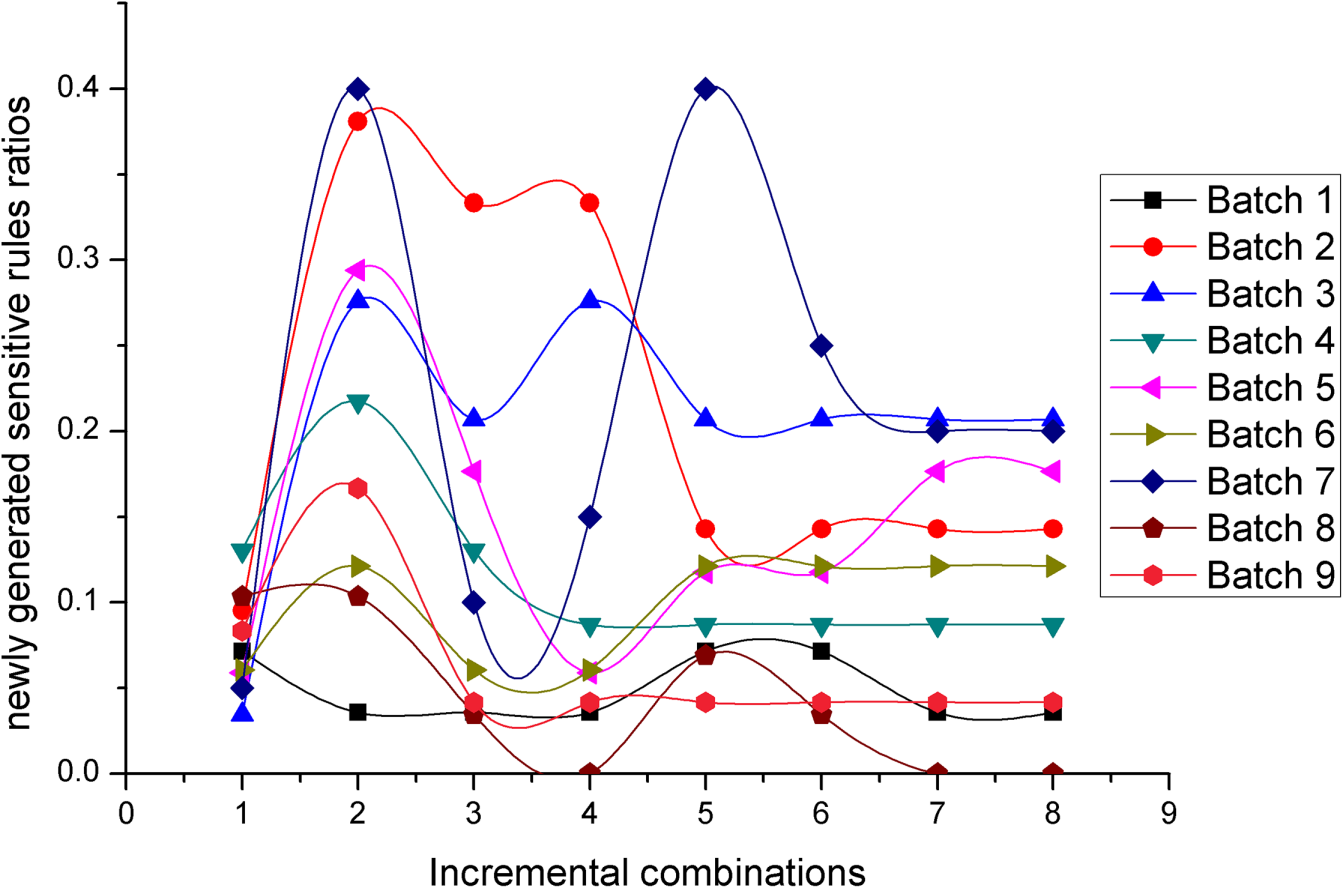
Newly generated sensitive rules ratios change with data expansion using the *tSTK* method.

Figs 12 and 13 present two measures in terms of newly generated sensitive rules ratios, for the *NOSTK* method and the *tSTK* method, respectively. The following can be seen from the comparison: (1)For the same batch of data, and the same incremental combination, the values for newly generated sensitive rules ratios based on the *NOSTK* method were all less than the values based on the *tSTK* method. (2) As combinations increased, the values for newly generated sensitive rules ratios based on the *NOSTK* algorithm could tend toward a lower range more quickly than the values from the *tSTK* method. Specifically, after an incremental combination was up to 5, for all batches of privacy-sensitive sequence rules, the range of the *NOSTK* algorithm was [0–0.117647059], while the range of the *tSTK* method was [0–0.4]. These experimental results showed that the *NOSTK* method as a whole, when compared to the *tSTK* method, had fewer side effects in terms of newly generated sensitive rules ratios. Moreover, the side effects that did arise could converge quickly to a smaller threshold range with data expansion by incremental combinations.

**Fig 14:**
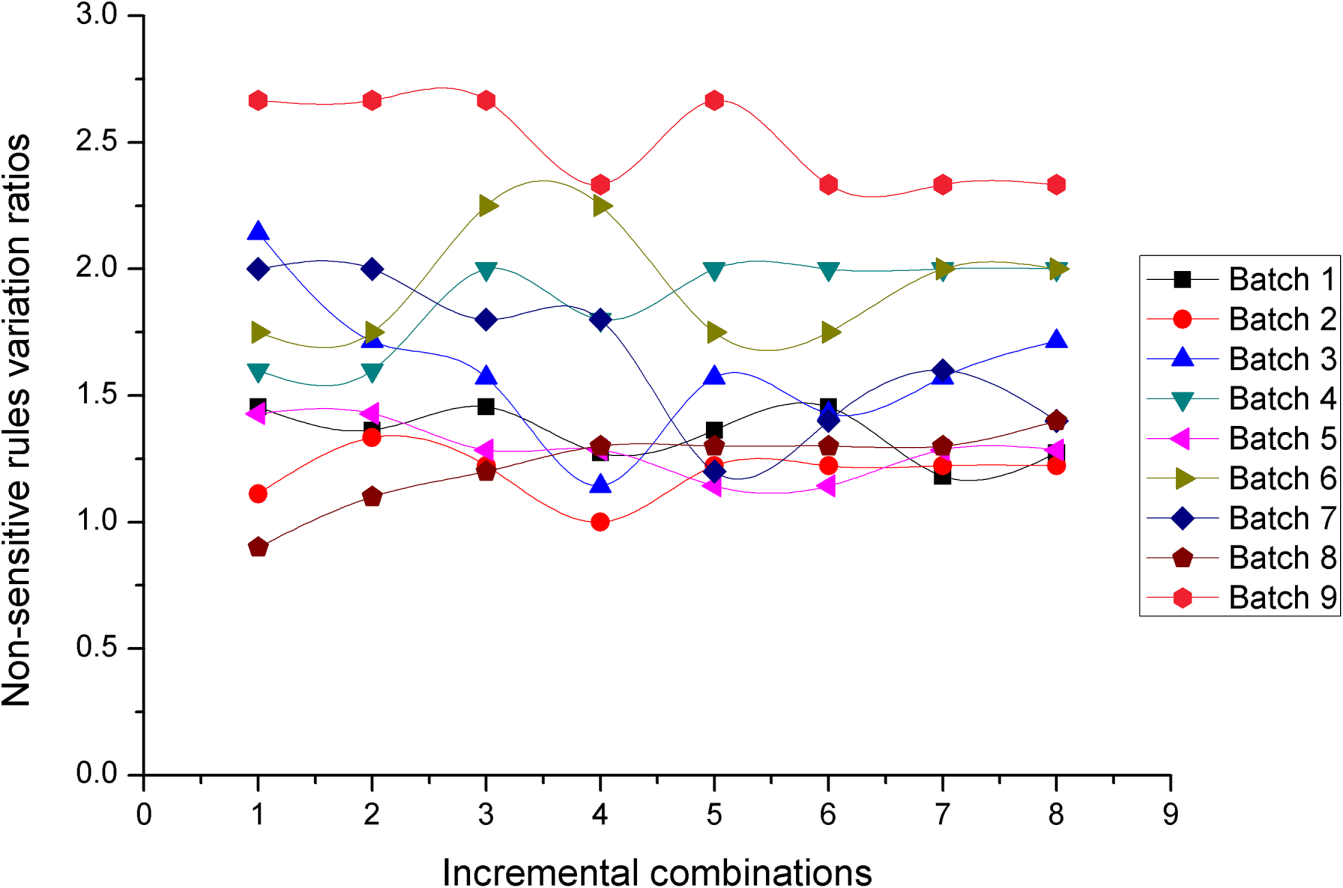
Non-sensitive rules variation ratios change with data expansion using the *NOSTK* method.

**Fig 15:**
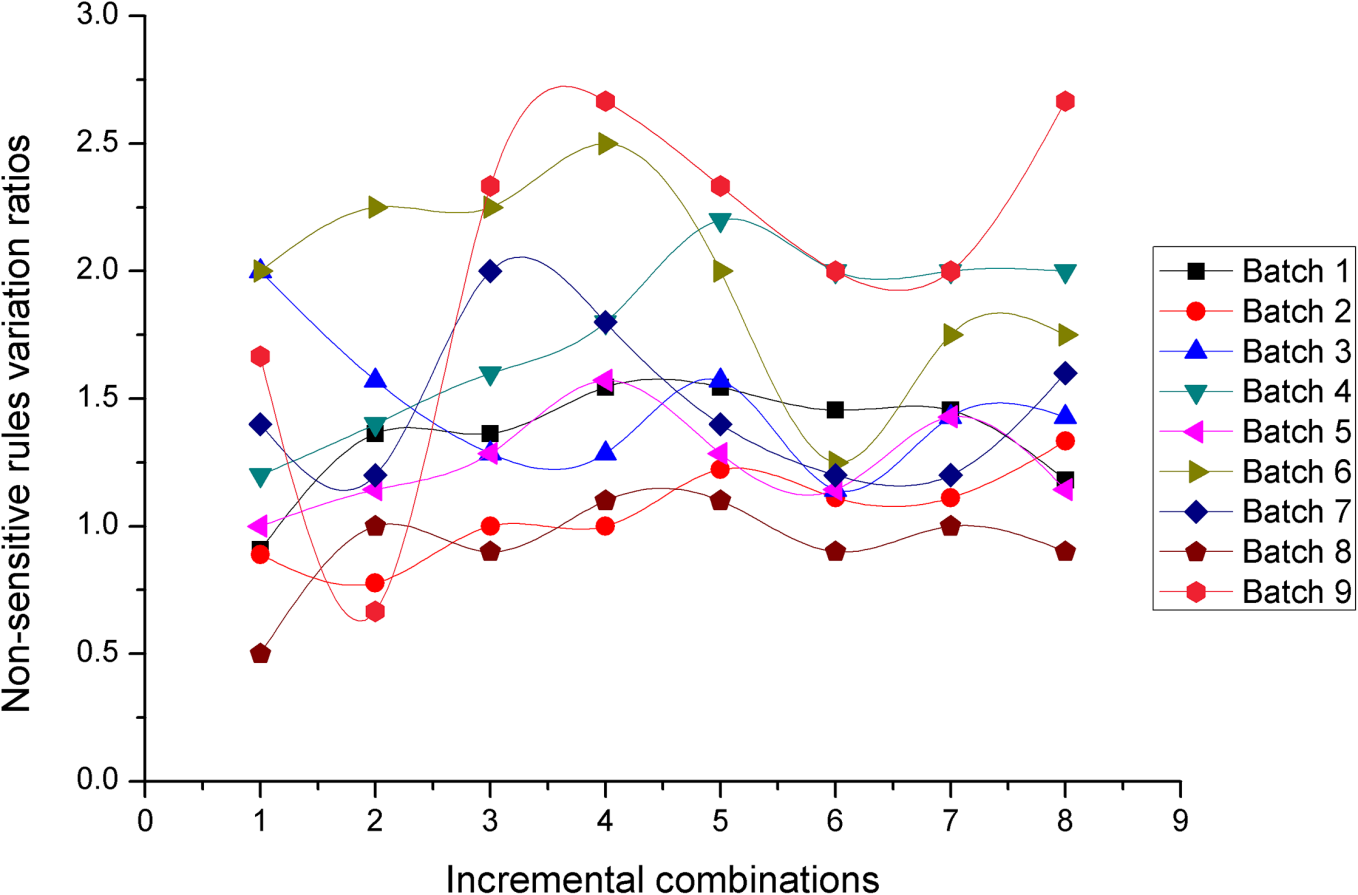
Non-sensitive rules variation ratios change with data expansion using the *tSTK* method.

**Fig 16:**
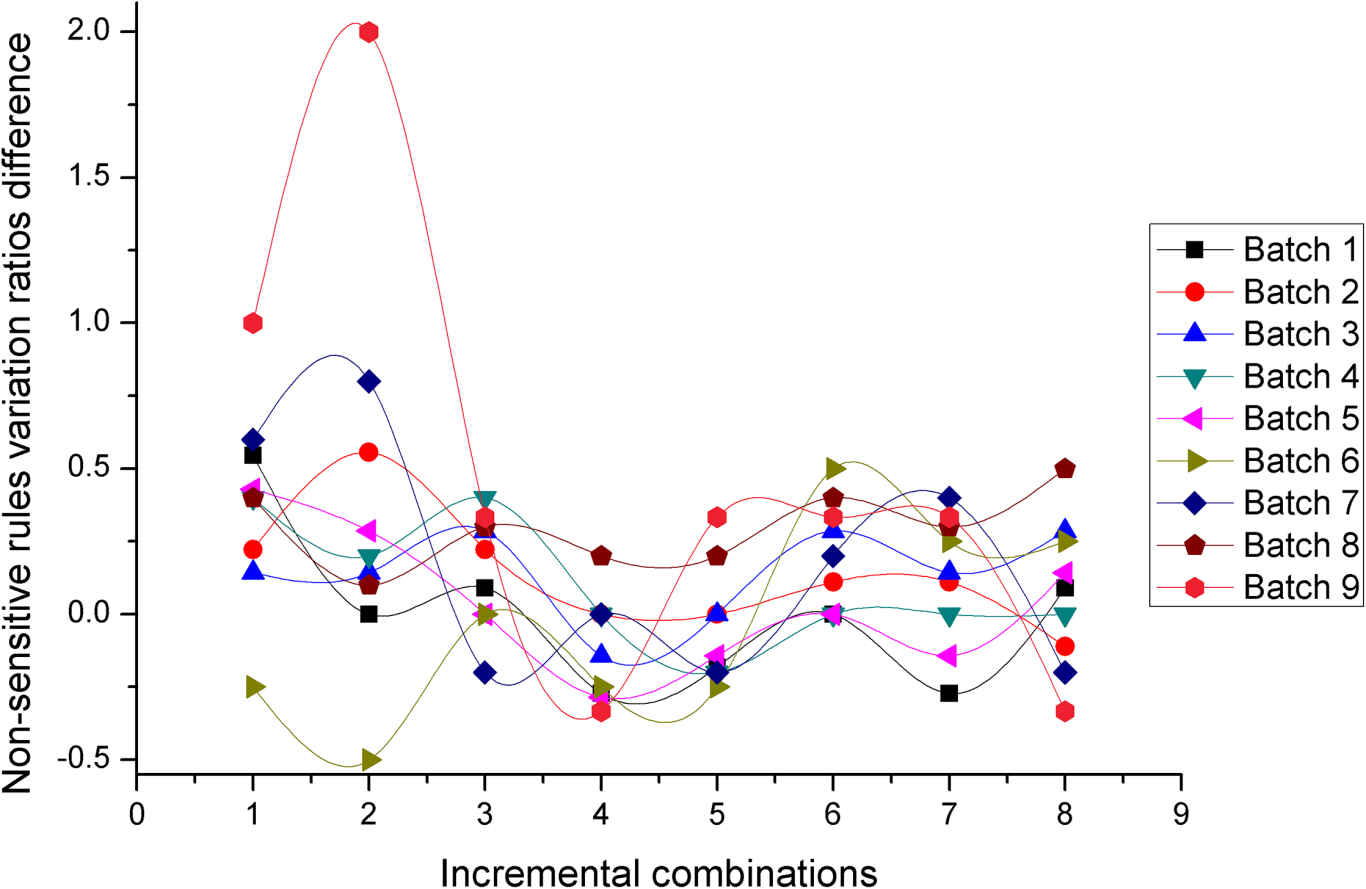
Non-sensitive rules variation ratios difference between the *NOSTK* method and the *tSTK* method.

Figs 14 and 15 present two measures in terms of non-sensitive rules variation ratios, for the *NOSTK* method and the *tSTK* method, respectively. The following can be seen from the comparison: (1) For 9 batches of data, whether using the *NOSTK* method or the *tSTK* method, ratios for non- sensitive rules variation fluctuated irregularly as combinations increased. (2) For a same batch of data, and a same incremental combination, the values for non- sensitive rules variation ratios based on the *NOSTK* method was basically the same as the ones based on the *tSTK* method, and not necessarily which value is greater. This point can be seen more clearly from Fig 16 (the difference between the values in Figs 14 and 15): as the data increment combinations were not less than 3, for the 9 batches of data, the difference values were fluctuating in the value of 0; as the data increment combinations were 1, 2, the difference values for batch 3 were less than 0, and the values for other batches were greater than or equal to 0.

These experimental results showed that there was no significant difference between the *NOSTK* method and the *tSTK* method in terms of non-sensitive rules variation ratios, which verified the theoretical analysis that the normal generalization principle and the minimum generalization principle help to maintain the utilization of non-privacy-sensitive sequence rules (in *Avoidance and generalization* section).

In summary, we found that the *NOSTK* method can hide privacy-sensitive sequence rules in terms of hiding sensitive rules ratios more quickly and effectively to eliminate inference attacks. The *NOSTK* method also had fewer side effects in terms of newly generated sensitive rules ratios than the *tSTK* method, and had basically the same side effects in terms of non-sensitive rules variation ratios with the *tSTK* method.

### Experiment 2: Performance variation with the parameter *K* of the *NOSTK* method

**Fig 17:**
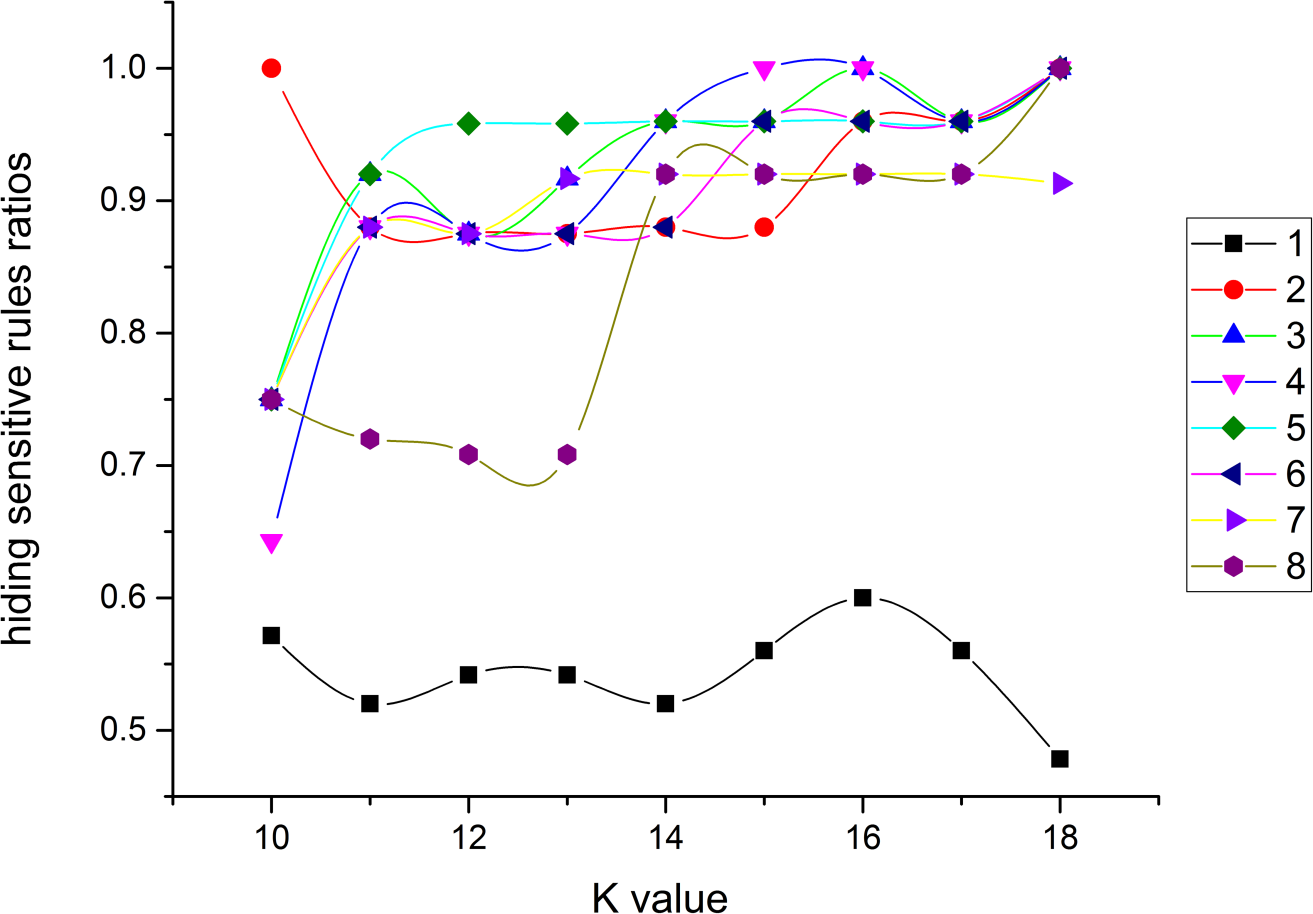
Hiding sensitive rules ratios change with *K* values for different incremental combinations.

**Fig 18:**
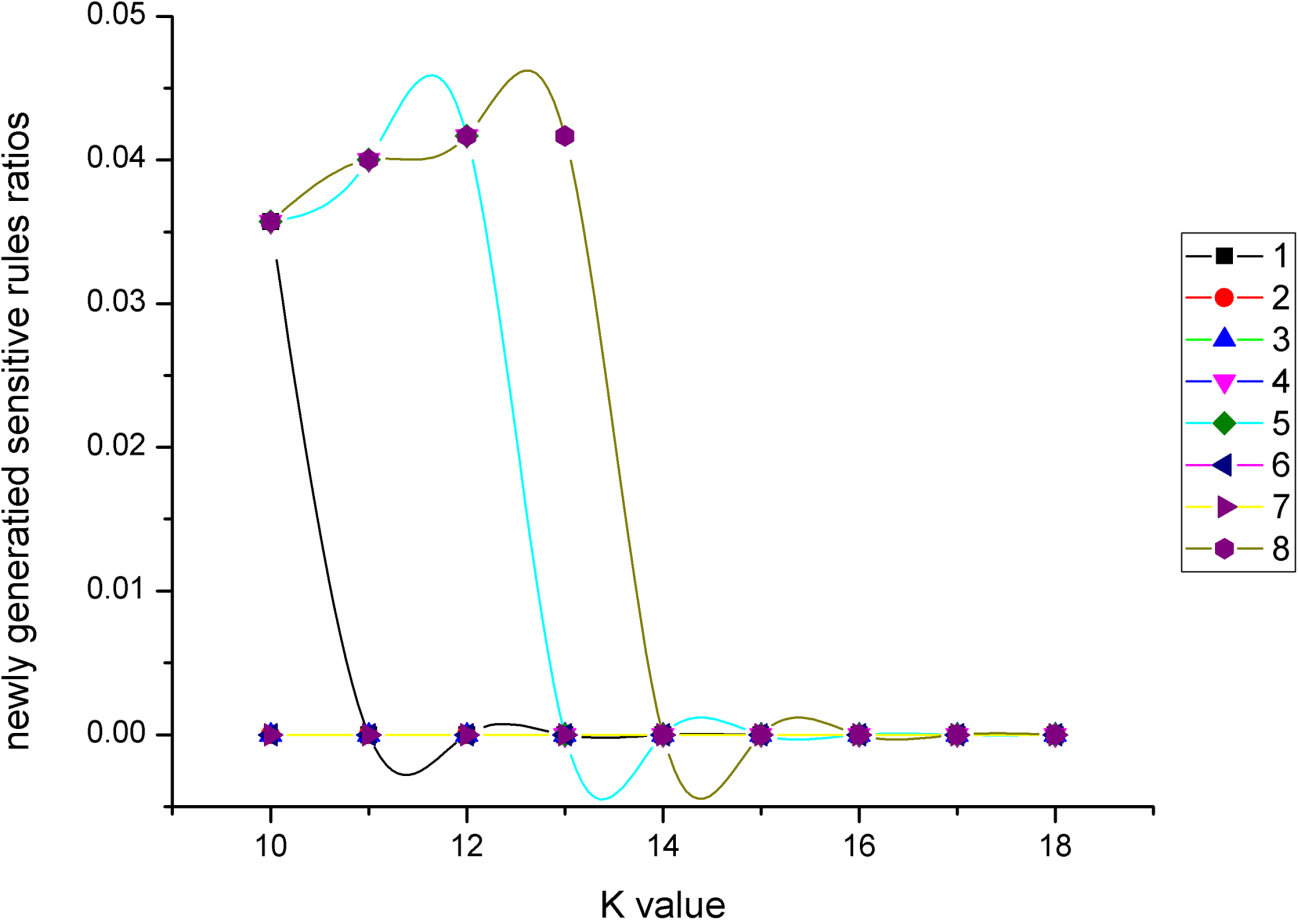
Newly generated sensitive rules ratios change with *K* values for different incremental combinations.

**Fig 19:**
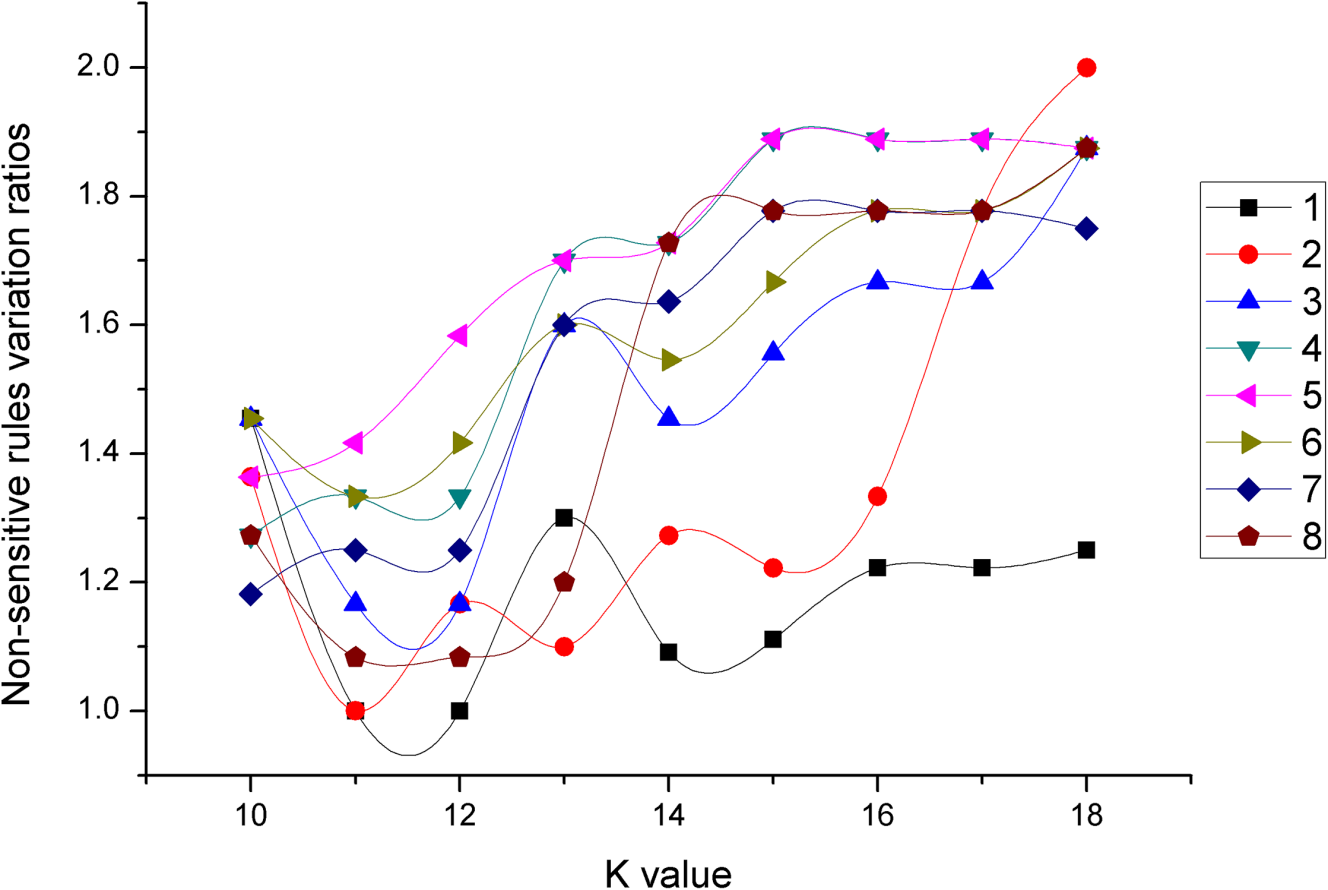
Non-sensitive rules variation ratios change with *K* values for different incremental combinations.

This experiment explored the performance variation of the *NOSTK* method on the sequence rules mined from 72 incremental combinations with different *K* values (*K* = 10~18). Figs 17–19 present the performance variation with the parameter *K* for the *NOSTK* algorithm for hiding sensitive rules ratios, newly generated sensitive rules ratios, and non-sensitive rules variation ratios, respectively.

As we can see from Fig 17, for each incremental combination, with the increase of *K* value, ratios for hiding sensitive rules first fluctuated slightly, and then increased and reached a peak value. Specifically, as the *K* value was 16, hiding sensitive rules ratios reached a peak.

As we can see from Fig 18, for all incremental combinations, with the increase of *K* value, newly generated sensitive rules ratios first began to increase slightly, and then quickly changed to be 0. For example, after the *K* value was 14, the newly generated sensitive rules ratios of all incremental combinations changed to 0.

As we can see from Fig 19, for all incremental combinations except for incremental combinations 1, with the increase of *K* value, non-sensitive rules variation ratios as a whole gradually increase.

**Fig 20:**
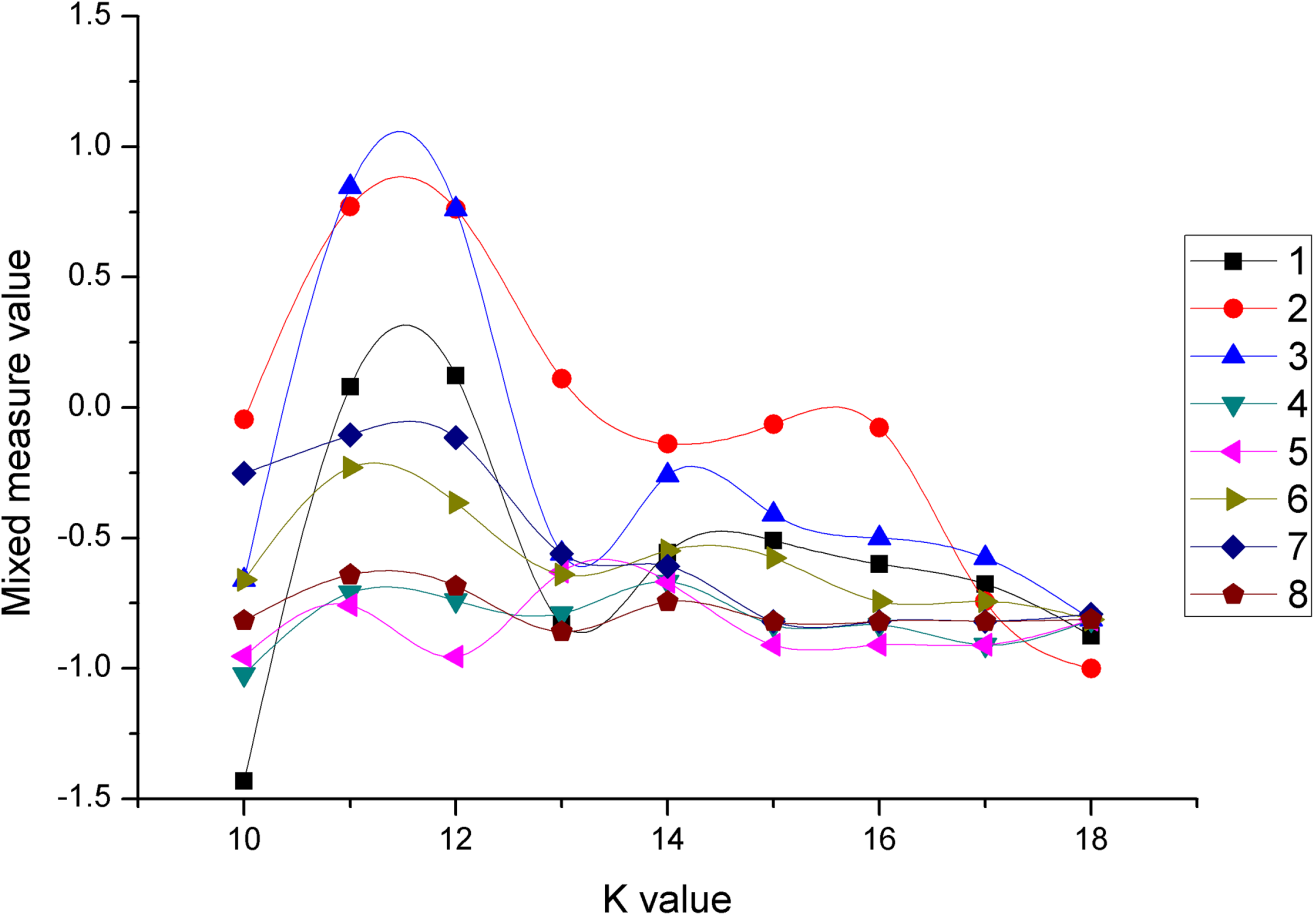
Mixed measure values change with *K* values for different incremental combinations.

According to the performance variation tendency of the *NOSTK* method with the parameter *K* value, we can conclude that choosing an appropriate *K* parameter value for generating anonymity datasets and selecting an appropriate incremental combination can help achieve the optimal goal of hiding the maximum number of original sensitive rules while generating a minimum number of new sensitive rules and affecting a minimum number of non-sensitive rules. To achieve this goal, we designed a mixed measure based on these three parameters: hiding sensitive rules ratios, newly generated sensitive rules ratios and non-sensitive rules variation ratios, denoted as *hsrr*, *ngsrr* and *nsrvr*, respectively. First, we used the Min-Max Normalization method formulated as $X^{*}=\frac{x-min}{max-min}$ to normalize the three parameters and got the processed results denoted as *hsrr*^*n*^, *ngsrr*^*n*^ and *nsrvr*^*n*^. Next, we designed a mixed measure formulated as $Y=hsrr^{n}-\frac{\left(ngsrr^{n}+nsrvr^{n}\right)}{2}$ and got the mixed measure values from Figs 17–19, which was shown as Fig 20. Finally, from Fig 20 we can see that for each specific incremental combination, there was a peak value of the mixed measure values as the *K* value increases. Specifically, for the incremental combination 2, 3, 6, 7 and 8, all the peak values appeared at a *K* value of 11; for the incremental combination 1, 4 and 5, the peak values appeared at a *K* value of 12, 14 and 13, respectively. Furthermore, we can get two typical optimization cases: (1) if the optimal mixed measure value was expected to be acquired quickly, the *K* value should be set to 12, and the optimal mixed measure value (0.121527558) was obtained when the incremental combination was 1. (2) if the time to obtain the optimal value is not limited, the optimal mixed measure value (0.846666628) was obtained when the *K* value was set to 11 and the incremental combination was 3.

## Conclusion

Traditional privacy defense techniques cannot prevent inference attacks based on privacy-sensitive sequence rules mined from large-scale anonymity datasets. To overcome this challenge, we defined formally a destination location prediction attack model, and then presented a countermeasure technique using a novel spatial temporal k-anonymity (*NOSTK*) method. We conducted extensive experiments to test the efficiency of the proposed method. The results demonstrated that our proposed method could hide sensitive rules more quickly and completely to eliminate inference attacks. Our method also had fewer side effects in terms of newly generated sensitive rules ratios than the traditional spatial-temporal k-anonymity (*tSTK*) method, and had basically the same side effects in terms of non-sensitive rules variation ratios with the *tSTK* method. In addition, we also found the performance variation tendency of the *NOSTK* method with the parameter *K* value, which can help achieve an optimal goal of hiding the maximum number of original sensitive rules while generating a minimum number of new sensitive rules and affecting a minimum number of non-sensitive rules.

As big data processing continues to be the target of sophisticated privacy attacks, research efforts will remain focused on implementation of data mining methods for large-scale anonymity datasets that can support privacy protection techniques [48,49]. The main direction of our future research will be to ensure that our proposed *NOSTK* method can be implemented on big data computing platforms to help resist attacks based on large-scale data mining.

## Supporting information

S1 Dataset Simulated sequences of cloaking regions (original) and sequence of query grid cells. (ZIP)

S2 Dataset Sequence rules mined from simulated sequences of cloaking regions (original). (ZIP)

S3 Dataset Simulated sequences of cloaking regions (expansion). (ZIP)

S4 Dataset Sequence rules mined from simulated sequences of cloaking regions (expansion). (ZIP)

S5 Dataset Performance evaluation metrics of the NOSTK and the tSTK methods. (ZIP)

S1 Text Introduction of datasets in experiments. (DOC)
