# Supplementary material for: A novel on-line spatial-temporal k-anonymity method for location privacy protection from sequence rules-based inference attacks
Source: PLoS One. 2017 Aug 2;12(8):e0182232. doi: 10.1371/journal.pone.0182232 (PMC5540611; doi:10.1371/journal.pone.0182232)
Supplement: S1 Text — (DOC) [file pone.0182232.s006.doc]

# Introduction of datasets in experiments

In this paper, minimal dataset was shown in folder *dataset*, and the details are shown in Table I~V.

Table I. Description of simulated sequences of cloaking regions (original) and sequence of query grid cells

| Folder path | Sub-Folder | File name | Description |
| --- | --- | --- | --- |
| ..\dataset\1 orginal\sequence\SCR\ | k=10~18 | Batch No 1.txt~Batch No 9.txt | 81 batches of sequences of cloaking regions with K=10~18 |
| ..\dataset\1 orginal\sequence\SQGC\ | k=10~18 | Batch No 1.txt~Batch No 9.txt | 81 batches of query grid cells with K=10~18 |

Table II. Description of sequence rules mined from simulated sequences of cloaking regions (original)

| Folder path | Sub-Folder | File name | Description |
| --- | --- | --- | --- |
| ..\dataset\1 orginal\rule\ | k=10~18 | Batch No 1.txt~Batch No 9.txt  PSR grid.txt | 81 batches of sequence rules mined from 81 batches simulated sequences of cloaking regions with K=10~18(Table I).  PSR grid cells for each batch of sequence rules. |

Table III. Description of simulated sequences of cloaking regions (expansion)

| Folder path | Sub-Folder | Sub-Folder | File name | Description |
| --- | --- | --- | --- | --- |
| ..\ dataset\2 expansion\sequence\Data expansion using tSTK\ | k=10 | For SeR 1 | 1_1.txt~1_8.txt | Simulated 72 batches of incremental combinations of sequences of cloaking regions generated by the tSTK method to hide batch No. 1~9 of privacy-sensitive sequence rules with K=10. |
|  |  | For SeR 2 | 2_1.txt~2_8.txt |  |
|  |  | For SeR 3 | 3_1.txt~3_8.txt |  |
|  |  | For SeR 4 | 4_1.txt~4_8.txt |  |
|  |  | For SeR 5 | 5_1.txt~5_8.txt |  |
|  |  | For SeR 6 | 6_1.txt~6_8.txt |  |
|  |  | For SeR 7 | 7_1.txt~7_8.txt |  |
|  |  | For SeR 8 | 8_1.txt~8_8.txt |  |
|  |  | For SeR 9 | 9_1.txt~9_8.txt |  |
| ..\dataset\2 expansion\sequence\Data expansion using NOSTK\ | k=10 | For SeR 1 | 1_1.txt~1_8.txt | Simulated 72 batches of incremental combinations of sequences of cloaking regions generated by the NOSTK method to hide batch No. 1~9 of privacy-sensitive sequence rules with K=10. |
|  |  | For SeR 2 | 2_1.txt~2_8.txt |  |
|  |  | For SeR 3 | 3_1.txt~3_8.txt |  |
|  |  | For SeR 4 | 4_1.txt~4_8.txt |  |
|  |  | For SeR 5 | 5_1.txt~5_8.txt |  |
|  |  | For SeR 6 | 6_1.txt~6_8.txt |  |
|  |  | For SeR 7 | 7_1.txt~7_8.txt |  |
|  |  | For SeR 8 | 8_1.txt~8_8.txt |  |
|  |  | For SeR 9 | 9_1.txt~9_8.txt |  |
|  | k=11~18 | For SeR 1 | 1_1.txt~1_8.txt | Simulated 64 batches of incremental combinations of sequences of cloaking regions generated by the NOSTK method to hide batch No. 1 of privacy-sensitive sequence rules with K=11~18. |

Table IV. Description of sequence rules mined from simulated sequences of cloaking regions (expansion)

| Folder path | Sub-Folder | Sub-Folder | File name | Description |
| --- | --- | --- | --- | --- |
| ..\dataset\2 expansion\rule\SeR with tSTK expansion\ | k=10 | For SeR 1 | 1_1.txt~1_8.txt | Mined 72 batches sequence rules from incremental combinations with K=10 using the tSTK method (Table III). |
|  |  | For SeR 2 | 2_1.txt~2_8.txt |  |
|  |  | For SeR 3 | 3_1.txt~3_8.txt |  |
|  |  | For SeR 4 | 4_1.txt~4_8.txt |  |
|  |  | For SeR 5 | 5_1.txt~5_8.txt |  |
|  |  | For SeR 6 | 6_1.txt~6_8.txt |  |
|  |  | For SeR 7 | 7_1.txt~7_8.txt |  |
|  |  | For SeR 8 | 8_1.txt~8_8.txt |  |
|  |  | For SeR 9 | 9_1.txt~9_8.txt |  |
| ..\dataset\2 expansion\rule\ SeR with NOSTK expansion \ | k=10 | For SeR 1 | 1_1.txt~1_8.txt | Mined 72 batches sequence rules from incremental combinations with K=10 using the NOSTK method (Table III). |
|  |  | For SeR 2 | 2_1.txt~2_8.txt |  |
|  |  | For SeR 3 | 3_1.txt~3_8.txt |  |
|  |  | For SeR 4 | 4_1.txt~4_8.txt |  |
|  |  | For SeR 5 | 5_1.txt~5_8.txt |  |
|  |  | For SeR 6 | 6_1.txt~6_8.txt |  |
|  |  | For SeR 7 | 7_1.txt~7_8.txt |  |
|  |  | For SeR 8 | 8_1.txt~8_8.txt |  |
|  |  | For SeR 9 | 9_1.txt~9_8.txt |  |
|  | k=11~18 | For SeR 1 | 1_1.txt~1_8.txt | Mined 64 batches sequence rules from incremental combinations with K=11~18 using the NOSTK method (Table III). |

Table V. Performance evaluation metrics of the *NOSTK* and the *tSTK* methods

| Folder path | Sub-Folder | Sub-Folder | File name | Description |
| --- | --- | --- | --- | --- |
| ..\dataset\3 comparison\Performance with tSTK | k=10 | For SeR 1~ For SeR 9 | result.txt | Performance evaluation metrics of hiding batch No. 1~9 of privacy-sensitive sequence rules with K=10 using the tSTK method, by comparing the corresponding sequence rules in Table II with the sequence rules in Table IV. |
| ..\dataset\3 comparison\Performance with NOSTK | k=10 | For SeR 1~ For SeR 9 | result.txt | Performance evaluation metrics of hiding batch No. 1~9 of privacy-sensitive sequence rules with K=10 using the NOSTK method, by comparing the corresponding sequence rules in Table II with the sequence rules in Table IV. |
|  | k=11~18 | For SeR 1 | result.txt | Performance evaluation metrics of hiding batch No. 1 of privacy-sensitive sequence rules with K=11~18 using the NOSTK method, by comparing the corresponding sequence rules in Table II with the sequence rules in Table IV. |
